# AceView: a comprehensive cDNA-supported gene and transcripts annotation

**DOI:** 10.1186/gb-2006-7-s1-s12

**Published:** 2006-08-07

**Authors:** Danielle Thierry-Mieg, Jean Thierry-Mieg

**Affiliations:** 1 National Center for Biotechnology Information, National Library of Medicine, NIH, Bethesda, MD 20894, USA

## Abstract

**Background:**

Regions covering one percent of the genome, selected by ENCODE for extensive analysis, were annotated by the HAVANA/Gencode group with high quality transcripts, thus defining a benchmark. The ENCODE Genome Annotation Assessment Project (EGASP) competition aimed at reproducing Gencode and finding new genes. The organizers evaluated the protein predictions in depth. We present a complementary analysis of the mRNAs, including alternative transcript variants.

**Results:**

We evaluate 25 gene tracks from the University of California Santa Cruz (UCSC) genome browser. We either distinguish or collapse the alternative splice variants, and compare the genomic coordinates of exons, introns and nucleotides. Whole mRNA models, seen as chains of introns, are sorted to find the best matching pairs, and compared so that each mRNA is used only once. At the mRNA level, AceView is by far the closest to Gencode: the vast majority of transcripts of the two methods, including alternative variants, are identical. At the protein level, however, due to a lack of experimental data, our predictions differ: Gencode annotates proteins in only 41% of the mRNAs whereas AceView does so in virtually all. We describe the driving principles of AceView, and how, by performing hand-supervised automatic annotation, we solve the combinatorial splicing problem and summarize all of GenBank, dbEST and RefSeq into a genome-wide non-redundant but comprehensive cDNA-supported transcriptome. AceView accuracy is now validated by Gencode.

**Conclusion:**

Relative to a consensus mRNA catalog constructed from all evidence-based annotations, Gencode and AceView have 81% and 84% sensitivity, and 74% and 73% specificity, respectively. This close agreement validates a richer view of the human transcriptome, with three to five times more transcripts than in UCSC Known Genes (sensitivity 28%), RefSeq (sensitivity 21%) or Ensembl (sensitivity 19%).

## Background

Annotating the genes, transcripts and proteins of the human genome is a significant challenge. How many genes will ultimately be identified, what mechanisms control transcription, alternative splicing, the stability of the transcripts, translatability, what role do non-coding genes play and are there identifiable signals encoded in the genome sequence that control these events are all questions that need to be resolved so that we can hope to annotate the human genome faithfully. To address this type of question, the ENCODE project [1], launched by the National Human Genome Research Institute, encourages a concentration of international efforts and expertise on 1% of the human genome, in 44 carefully selected regions taken as representative of the whole genome, in the hope that mature annotation techniques will be developed, validated, and further applied to the entire genome.

The UCSC genome browser [2] provides fast and open access to a highly configurable view of a wealth of sequence-based genome annotations. The evidence-based or predicted gene tracks are an open repository for genome-wide annotations of the genes, and most tracks are well documented. All the data can easily be retrieved in a uniform format. The submission process is also simple and friendly, and there are no signs of limits to the amount of data that can be displayed and distributed by this group: the UCSC genome browser was naturally selected as the official repository for sequence-related data for the ENCODE project [3].

The Human and Vertebrate Analysis and Annotation (HAVANA) teams are expert at manual gene annotation [4]. They "require that all annotated gene structures (transcripts) are supported by transcriptional evidence, either from cDNA, expressed sequence tag (EST) or protein sequences, and as such not all annotated transcripts are necessarily complete". They typically bring to the curator, in a specialized Acedb-based display, a combination of evidence from alignment of mRNAs, ESTs and proteins, from human and other vertebrates. Curators hand select the best supported transcript models, and occasionally experimentally extend or confirm a model, using reverse transcription polymerase chain reaction and/or rapid amplification of cDNA ends. In this way, the Sanger Institute group carefully annotated the 44 ENCODE regions. Their gene models on these regions are called Gencode. They identify five times more variants than RefSeq, yet all their transcripts should be considered experimentally validated.

The ENCODE gene annotation assessment project (EGASP) [5,6] launched a competition among gene-predicting programs to try to best reproduce the Gencode annotations, taken as a reference, and/or to predict novel transcripts; the most promising novel genes would eventually be validated by RT-PCR. The Gencode solutions for 13 training regions were released at the end of 2004, and interested parties were asked to annotate the remaining 31 test regions before the solutions were unveiled in May 2005. Sixteen teams contributed complete mRNA or protein models; AceView was one of them.

The AceView program [7], developed at NCBI, provides a strictly cDNA-supported view of the human transcriptome and the genes by summarizing all quality-filtered human cDNA data from GenBank, dbEST and the RefSeq. The nematode version (also known as WormGenes) is even more evolved and heavily hand curated: it uses over 280,000 cDNA sequencing traces, provided by the Kohara laboratory (Y Kohara, T Shin-i, Y Suzuki, S Sugano, D Thierry-Mieg and J Thierry-Mieg, personal communication) and the worm community, that we hand edit and use as a training set to handle automatically EST sequence basecall errors. AceView was written from scratch and guided over the years by visual expert evaluation and users' reports; it uses heuristics to closely reproduce manual curation in an automatic way. Annotation is a difficult and dynamic problem, and we do not claim to have a final solution, yet we hope to stimulate experiments and accelerate discovery. Our results are frequently updated as new cDNA sequences are submitted to the nucleotide databases, and they have been publicly available at NCBI since 2000 [7]. AceView, previously called Acembly, is also displayed as one of the UCSC gene tracks and as a DAS track on the Ensembl browser.

We submitted to EGASP a lightly hand edited version of the public AceView, with the note: "AceView: All mRNAs and cDNAs available in GenBank, excluding NMs, were co-aligned on the Gencode regions. The results were then examined and filtered to resemble HAVANA. The very restrictive view of HAVANA on CDS was not reproduced, due to a lack of experimental data." Our special treatment consisted of eliminating single exon genes, unless they had a Pfam annotation (as an unfortunate consequence, we lost a number of olfactory receptors correctly represented in the standard AceView) and discarding the RefSeqs, to avoid second-hand annotation. We also removed several recent retroposon-type pseudogenes that had escaped our standard filters and about 50 cDNA clones aligning with non-standard introns (not GT/AG or GC/AG). Then, after the workshop, we modified our program to automatically perform some of this extra filtering: the current genome-wide public AceView, dated August 2005, benefits from these Gencode-driven improvements and rates even slightly better than the EGASP version. Numbers quoted in the text correspond to the genome-wide version, because it is more relevant to the AceView users, but the closely similar AceView/EGASP performances are displayed graphically and in numbers in Additional data files 2-4.

In the main EGASP paper, Guigo *et al*. [6] thoroughly analyze the novel gene predictions and all regions annotated as protein coding by Gencode; they also present some basic comparisons of the mRNAs. Their preferred mode is to project on the genome the features to be compared, for instance nucleotides or exons, and to count each element only once per gene, in a way flattening the alternative transcript variants. However, as Gencode indicates, human gene transcription and splicing patterns are complex, yet not combinatorial, and the exon-intron chaining cannot be rendered in projection. We therefore undertook a complementary analysis of the same data, but focused on the complete mRNAs, irrespective of whether or not they have an annotated coding sequence (CDS). We took special interest in comparing the alternative variants across the tracks, using the complete chains of introns as signatures. We wrote a standalone program, UCSCtrackCompare, to compare the models of the various tracks to the Gencode validated transcripts (October 2005 freeze).

In general agreement with Table 6 from [6] for the projected view, but much more clearly when we look separately at the alternative variants, we show that at the mRNA level, AceView transcripts are by far the closest match to Gencode transcripts: all nucleotides used in spliced variants are common, except for 8% specific to Gencode or 12% specific to AceView. All introns are common, except for 10% specific to Gencode or 14% specific to AceView. In contrast, due to a lack of large scale protein sequence evidence, we have chosen different strategies to annotate the proteins: Gencode proteins (of which 31% are partial) are annotated in only 41% of the transcripts. It does not necessarily mean that HAVANA predicts that the remaining 59% transcripts are non-coding, but this is definitely what the Guigo *et al*. analysis [6] assumed, and this biased their CDS analysis. AceView, like all other methods except Gencode, conservatively chose to annotate the best predicted CDS in nearly all transcripts. Notice also that the Gencode hand annotation of transcripts is available at this high quality only in the ENCODE regions: their annotation of chromosome 20, for example, is far less comprehensive (Thierry-Mieg and Thierry-Mieg, unpublished) [4,7]; it may be too time consuming to annotate a whole chromosome manually at this depth while the cDNA data are accumulating so fast. In contrast, because AceView is automatically generated, it can provide for the whole genome a regularly updated annotation of the intron-exon structure of the genes and their alternative transcript variants that, as we show here, is of a quality comparable to the manual Gencode annotation. If deemed desirable, AceView mRNAs could easily be re-annotated with parsimonious Gencode-like CDSs.

The excellent agreement in gene structure between Gencode and AceView provides a cross validation of both annotations. The cDNA-supported consensus transcriptome includes close to five times more transcripts than RefSeq, and three times more than UCSC 'Known Genes', a track summarizing the human protein-coding quality-filtered data from RefSeq, GenBank mRNAs, and UniProt.

## Results and discussion

### Comparison of gene models using the UCSCtrackCompare program

To compare the performance of all programs at reproducing the rich Gencode annotation, we wrote a standalone program, UCSCtrackCompare, available in source form in Additional data file 1. The program compares, in chosen genomic regions, a UCSC track or a collection of tracks taken as reference (usually Gencode) against any number of selected tracks. It produces, in about 10 minutes, support for the analysis presented here; for a more detailed comparison of transcripts see Additional data file 2 and for coding regions see Additional data file 3. The direct outputs of UCSCtrackCompare were pasted in an Excel document (Additional data file 4). For the analyses of coding regions comparable to [6], our results almost exactly match those obtained by Guigo *et al*., and our results lie within 0.2% of theirs (Table 4 and 5 in [6]). However, there are uneven discrepancies that cannot be rationalized, but that may reach up to 8% in sensitivity and 13% in specificity, between our mRNA comparisons (Table 6 in [6]; see Additional data file 2.1). Methods that show an advantage in [6] include Ensembl, Exogean and Pairagon, and methods that show a disadvantage include AceView, ECgene, SGP2 and eight others. Yet, the general ordering of the methods is consistent across the two evaluations.

On 14 December 2005, we downloaded from UCSC all tracks with gene models in the 31 ENCODE test regions (see Additional data file 2.1). We selected for comparison to Gencode (October 2005 freeze) 14 non-redundant EGASP tracks released before the solutions, and 10 genome-wide tracks, distinguished on all diagrams by addition of an asterisk in front of the track name. Nine tracks, labeled P in front of their names, predict protein-only models by using *ab initio *methods, often integrating evolutionary sequence conservation; all others also use, or only use, mRNA and/or EST evidence. Statistics of the 25 tracks and hints on their inputs are summarized in Table 1.

Since the UCSC files give directly the exons of all tracks in a uniform chromosome-based coordinate system, the comparison of coordinates is straightforward and easy to duplicate (Additional data file 1). The choices offered in our program are either to decompose the models into their elements, exons, introns and nucleotides, or to consider them in their entirety; and then either to count each element with given genomic coordinates only once, thereby providing a rationalized projected measure of the unique elements (as was done in [6]), or to count each element as many times as it occurs in the alternative variants, providing a quantitative appraisal of the biological complexity of the gene. The two measures are complementary. Another option is to compare only the part of the models annotated as protein-coding. When applied to identical transcripts, this option allows the preferred hypotheses on choice of CDS and Start codon to be reverse engineered (Additional data file 2.5).

### Comparison of introns, exons and nucleotides in whole models

Consistently, when we compare whole mRNA models (rather than CDSs) to the Gencode reference, AceView fares remarkably well, better than any other track. This is true in the projected mode, and even more striking in the quantitative mode, where alternative variants are counted separately (see details in Additional data files 2 and 4).

**Figure 1:**
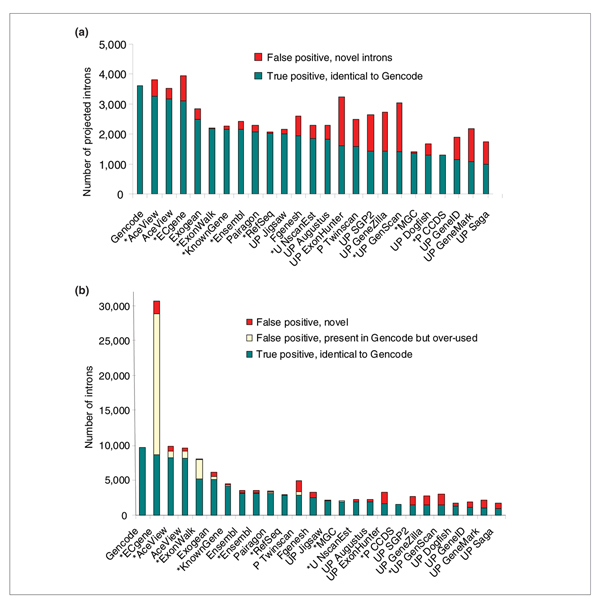
Comparison of introns between the Gencode reference and the 24 tracks, ordered by decreasing sensitivity, over the 31 test regions. Gencode validates 3,618 unique introns and a total of 9,693 introns in its alternative transcripts. **(a) **Projected measure: each intron is counted only once per method. Introns with the same coordinates as Gencode introns are shown in green and novel introns in red. The Gencode introns missed in each track (false negative) correspond to the distance between the 'true positive' bar and the Gencode reference, but are not explicitly represented. **(b) **Quantitative measure: all alternative variants are counted separately. Introns identical to Gencode introns, but over-used relative to Gencode are counted (in yellow) separately from novel introns that are not known to Gencode.

Nucleotides provide a global appraisal of the transcribed regions: AceView and Gencode spliced transcripts cover almost exactly the same nucleotides in the genome (92% sensitivity, 88% specificity; Additional data file 2.2). The structural precision of the models is best defined by the exact position of intron-exon boundaries. As shown in Figure 1a, most of the unique Gencode introns are used in AceView and few are added (sensitivity 90%, specificity 86%). Only AceView and ECgene (86.5%) detect more than 70% of the introns: the other EGASP tracks probably do not succeed in aligning with sufficient precision all the available ESTs and mRNAs. For reference, on 14 December 2005, RefSeq sees 56% of the Gencode validated introns and the Mammalian Gene Collection (MGC) 38%. As expected, the tracks relying mostly on *ab initio *predictions detect less Gencode introns, but more novel intron candidates, usually unique to each program (Additional data file 2.4); ExonHunter and Genscan are the most creative.

If all introns from alternative variants are counted separately (Figure 1b), Gencode uses close to three times the number of unique introns. AceView does too, but remarkably in this expansion, most of the introns remain exactly the same as in Gencode (sensitivity 85%, specificity 84%). In contrast, the specificity of ECgene drops to 28%, because of their drastic combinatorial use of the Gencode introns. This over-use feature is apparent to a lesser extent in the ExonWalk track, possibly because of excessive use of partial cDNAs in their step 3 (for documentation, see [3]).

The comparison of exons (Additional data file 2.3) can also assess the intron-exon boundaries of the models, but it is dominated by the effects of terminal exons, which represent 20% to 42% of all exons (depending on the method), and are often partial in transcript models. Furthermore, in contrast to intron boundaries, which are accurate and can in principle be verified by PCR or microarray experiments, the boundaries of terminal exons cannot be defined precisely even in a truly complete transcript because, biologically, the first (capped) base and the polyA addition site fluctuate *in vivo *[8] (D Thierry-Mieg and J Thierry-Mieg unpublished observations). Indeed, when we compare exons between Gencode and any other track, both sensitivity and specificity drop because of the terminal exons (Additional data file 2.3).

Another advantage of comparing spliced models through introns is that methods that predict only coding regions (labeled P(name) in all figures and tables) are less disadvantaged in intron than in exon mode, because most introns are located in the coding regions. This is true for example for 92% (2,075/2,264) of the unique introns in the UCSC 'Known Gene' track. We therefore chose to perform intron-based comparisons of whole spliced transcripts. Single exon genes and transcripts will be discussed separately below.

### N to N comparison of entire mRNAs across methods proposing alternative transcript models

**Figure 2:**
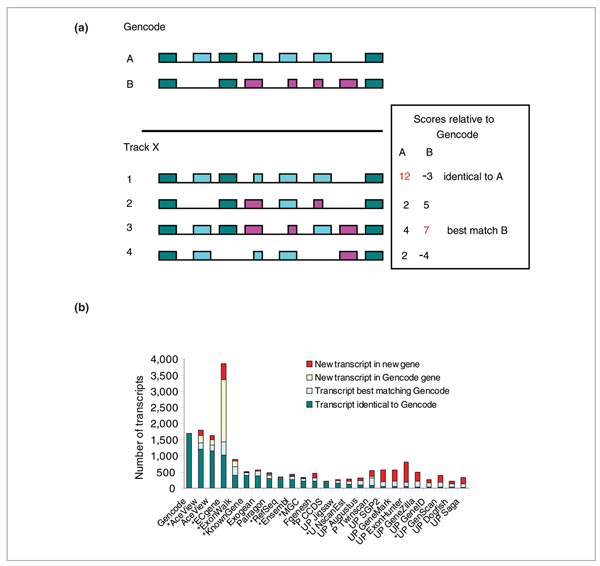
Comparison of whole transcripts. **(a) **Strategy for selecting the best one to one matching pairs. **(b) **Comparison of whole transcripts through their intron signatures. The number of transcripts identical to Gencode, best-matching but different from Gencode, new transcripts in Gencode genes and new transcripts in new genes are represented.

On average, Gencode genes with introns have 4.5 transcripts per gene; each transcript has 5.7 introns, but in projection only contributes 2.1 unique introns. If we limit this to coding transcripts, coding genes have on average 2.6 annotated CDSs, and each CDS has 8 introns, but in projection only contributes 3.7 unique introns (Additional data file 4, SummaryStats). Just a few of the possible combinations of introns correspond to supported models: this is called the combinatorial splicing problem. To properly compare performance across the tracks, we need to evaluate how the introns and exons are chained in the models. Let us consider each model as a non-separable chain of introns, the set of coordinates of its intron boundaries on the genome provides a precise signature. As illustrated in Figure 2a, we define the one-to-one best matching Gencode-to-track-X model pair by comparing all pairs and scoring intron boundaries: each boundary counts +1 if it is shared by the two models or -1 if it is unique to either of the two. In this way, identical models always score best. All pairs of models with one intron boundary in common are listed and sorted, and the two models from the best rating pair are flagged as 'best match', irrespective of their score. We then look recursively for the next best rating pair where none of the two models are flagged, and flag them. The remaining unflagged models in the list become 'additional variants in Gencode genes'. Finally, the models that do not occur in the list are orphans and belong to new genes, specific of method X, or to missed genes, present only in the Gencode reference. Some of the 'best matches' are actually 'identical to Gencode' in terms of intron-exon structure.

Figure 2b shows the results of this analysis (see also Additional data file 4, complete models). More than 70% of the Gencode transcripts have an exact structural equivalent in AceView, from the first to the last intron (1,191/1,691 = 70% sensitivity, 67% specificity); an additional 12% (206) have a best match. AceView misses 210 of the Gencode variants but sees 225 new variants in other Gencode genes. Finally, AceView misses some Gencode genes containing a total of 88 transcripts with introns, but sees some genes missed by Gencode, containing 170 transcripts with introns.

The second most sensitive track is ECgene (60% sensitivity; 26% specificity), which has twice as many spliced models as Gencode to choose from, but has fewer identical transcripts than AceView. The next best, ExonWalk and 'Known Genes', drop sharply to 23% exact matches. RefSeq sees only 17% of the Gencode transcripts and covers 53% of the unique nucleotides and 56% of the unique introns: despite its well recognized quality, RefSeq does not provide a comprehensive representation of the transcriptome.

### Intronless transcripts

By definition, the above analysis only evaluated transcripts with introns. Transcripts and genes without introns are in fact rare in the Gencode annotation (2.3% of the models, plus 1.7% due to clipping at the boundaries of the ENCODE regions). To better mimic Gencode, most were filtered by the EGASP participants, including AceView, which normally annotates a large number of single exon genes (Table 1; Additional data file 4).

Although rare on the entire UCSC browser, unspliced transcripts appear to be an important part of the human transcriptome, and they are an order of magnitude more frequent in mammals than in simpler Metazoa. Indeed, we compared the high quality full-length cDNA libraries made by Sugano using the oligo-capping method in either the nematode *Caenorhabditis elegans *or human (all sequences are in GenBank [7,9]). We removed 1.1% of clones that may be genomic contaminants (331/29,562 aligned in an intron-less gene ending on an A-rich region in the genome) and found that the percentage of fully sequenced intronless clones is 10 times greater in human than in worm (36% in human (10,578/29,562 FLJ clones) versus 3.5% in worm (2,010/56,671 worm yk capped clones)). These clones also map in 11 times more unspliced genes (with no spliced variants) in human than in worm (25% of these FLJ-containing genes in human (4,261/17,214 genes) versus 2% in worm (155/7,223 genes)). However, the level of possibly immature transcripts (unspliced, but from a gene with introns) is similar in the libraries from both species (57% (6,043/10,578) in human, 59% (1,187/2,010) in the worm).

According to our analysis, the huge increase in intronless genes is a major difference between the worm and human transcriptomes. It may have co-evolved with the increased usage of alternative splicing, increased intron length or other transcriptional features. An intronless transcript is not associated with an exon junction complex, so it is expected to be translated and degraded less efficiently in human [10-12], and it might be submitted to less evolutionary pressure: unspliced genes could be where new functions arise. Indeed, some of the unspliced genes potentially encode small proteins (18,385 intronless genes have hypothetical CDS of more than 100 amino acids; note that most are human or mammal specific), some may be regulatory non-coding RNAs, and some may just be transcription by-products. We do annotate these genes in the public AceView [7] and expect that in the future the role of intronless genes will be better apprehended.

### Are Gencode mRNAs fully validated and complete?

We compared the introns of Gencode to those of other tracks (Additional data file 2.4); 189 unique projected introns (5% of their 3,618) are seen exclusively in the Gencode transcripts but in no other track. We expect those to be supported by Gencode experimental validation, although the evidence was not submitted to GenBank as of August 2005. Another possibility is that some might be supported only by homology to non-human transcripts, as described in the standard HAVANA procedure [4], in which case we hope they are not exported to UniProt. Conversely, 681 'consensual' introns are seen by three tracks or more, or 340 are seen by the strictly cDNA-supported AceView that are not seen by Gencode. Actually, Gencode sees 8% of the consensual introns missed by AceView, but AceView sees 72% of the consensual introns missed by Gencode.

Overall, we find that the Gencode transcript annotation is quite comprehensive except for the quasi-absence of intronless genes. We confirm its high quality: it missed or annotated as pseudogene only a few expressed genes seen by AceView, and it did not exploit in the order of 15% of the introns represented in cDNAs from the public databases.

In general, we especially appreciate the fact that they report all observed transcripts with good alignments and standard introns, without filtering those that are structurally candidates for nonsense mediated RNA decay (NMD) [12]. According to our estimates [7], putative nonsense transcripts represent about 13% of the fully supported transcripts with predicted proteins of more than 100 amino acids in human (12,855 of 101,877 have introns larger than 60 base-pairs (bp) and with standard boundaries lying at least 55 bp downstream of the Stop codon), in contrast to 4% in worm (671 of 15,119 using the same criteria as above, except that minimal intron size is 30 bp). This increase may parallel the evolution of NMD into an essential multifunctional mechanism in mammals [10-12], or it may indicate that our cells have a more tolerant life style than the worm. Over the past few years, evidence that mRNA may be functionally active beyond its protein coding ability has accumulated [13]; human transcripts have a complex life, with mechanisms present to protect the mRNA, modulate its accessibility to the ribosome or to specific modification or processing enzymes, monitor its aging, its position in the cell, or its stability [10]. A comprehensive uninterpreted catalog of observed transcripts is needed to help understand all this complexity, and Gencode or AceView aim at this goal.

### Selecting protein coding transcripts and regions can only be an educated guess

A glimpse at the ENCODE regions of the UCSC genome browser [3] shows that Gencode and AceView transcripts look quite different from the other tracks, but so similar to one another that it would be hard to guess which is which if the names of the tracks were masked. However, Guigo and Reese [5] state that there is no clear winner at finding the Gencode coding regions. These two observations are in fact not contradictory: annotation of transcripts is based on large amounts of experimental cDNA evidence, so Gencode and AceView can agree almost perfectly. In contrast, we diverge on protein annotation, because there is almost no experimental protein sequence data available today. For this reason, most protein annotation remains hypothetical. Even proteins from UniProt/SwissProt are now contaminated by CDS predictions derived from transcriptome annotation, which makes new predictions by homology more and more circular. For instance, UniProt currently harbors 23,298 *C. elegans *proteins, but one should be aware that those are predictions: at most, 9,487 have complete cDNA support, and almost none has been sequenced. Until a substantial amount of direct protein sequences is generated, it is not surprising that different points of view coexist. For instance, Gencode annotates a CDS in only 41% of their transcripts, whereas all other tracks, including AceView, conventionally annotate a CDS in almost all their transcripts. Precisely because Gencode and AceView transcripts are so similar, the apparent specificity of AceView automatically drops by 59% when we compare CDSs, and the resemblance between the two methods is no longer striking (Additional data file 3, in agreement with [6] for the projected view).

In this context, AceView considers the CDS problem fully open, and offers no guarantee on which actual proteins are made [7]. In practice, we identify all possible CDSs, usually more than one per transcript, and annotate those larger than 50 amino acids using BlastP, PFAM, and Psort2. All hypothetical CDSs are available from our download page to help identify mass spectra. But to simplify the display, we pick a single 'best product' per transcript, knowing it may not reflect the situation *in vivo*, since it does not follow closely the rules indicated by Kozak [14]. For example, we do not necessarily choose the first CDS, which is quite often a short upstream open reading frame (uORF) [15,16], and we do not reinitiate and display multiple products per transcript. The 'best' protein is defined by considering, in a graded fashion, the presence of a Pfam protein domain, BlastP homologies, TaxBlast conservation, specific Psort annotations, maximization of introns within the CDS, position along the transcript and size of the CDS. If the mRNA is not known to be complete (if its 5' end is not defined by a capped clone) and the frame is open on the 5' side, the AceView CDS starts at the first in frame codon. But if the mRNA appears to be complete, the CDS starts at the first AUG codon, unless there is, in the correct environment, an in frame NUG or ANG codon [14,17-20] at least 180 bp upstream of the first AUG. In such a case, we annotate a predicted CDS starting at the non-AUG codon (the limit was set at 60 bp in the August 2005 release, leading to an excessive 24% complete CDS starting on an NUG start codon). *Escherichia coli *is reported to use about 17% non-AUG start, and to our surprise 7% of the human best complete products in AceView (20,616 of 293,158) actually have an alternative Start codon in the correct environment 60 amino acids upstream of the first AUG. It will be interesting to see how many are occasionally used as Start *in vivo*.

On the other hand, Gencode departs from all other programs in that it does not call a CDS unless it is conserved or already annotated in SwissProt, and it has a 'sensible' gene structure that is not a candidate for NMD. The product they annotate is almost always the same as AceView, except that the Start codon may differ. In reality, they probably do not really mean that 59% of the transcripts from protein coding genes are non-coding, but they just have to be very careful, because their proteins are poured directly in the UniProt reference database, so any annotation error will spread. We respect their attitude, yet some of their choices can be questioned.

NMD transcripts for instance are expected to produce, briefly but efficiently, truncated proteins, some of which could be functional [12]. Indeed, activation of mRNA degradation by this pathway requires a pioneering round of translation that, due to the dual role of NMD proteins in activating translation, should be very efficient on transcripts still decorated with exon junction complexes (reviewed in [10,11]). Furthermore, NMD is only shortening transcripts lifetime by 1.5 to 11 times [21], and it remains possible that it does not act in all cells and tissues at all times. The leaky behavior of the surveillance machinery is well known to geneticists: if no protein was produced from NMD candidates, the great majority of nonsense mutations would behave as complete loss of function (true nulls), but there are a number of counter-examples where a well positioned stop mutation leads to a gain of function phenotype (for example, *lin-1 *allele n1790 [22]).

With respect to the choice of the initiation codon, Gencode and other groups give much weight to interspecies conservation, they do not annotate upstream ORFs, and consider only AUG codons. But it is difficult to conceive how the ribosomes would be aware of these rules, instead of following the scanning mechanism experimentally established by Kozak [14]. If the transcript is accessible, upon scanning, the ribosome subunits assemble at the first AUG (or more rarely at an alternative start), irrespective of the length and inter-species conservation of the protein. If there is a stop codon soon after the AUG, the ribosome will keep scanning rather than drop off, and may reinitiate synthesis of a second product.

In summary, protein annotation is not supported by enough direct protein sequence evidence, and large scale mass spectrometry data on proteins are badly needed to clarify what happens *in vivo*.

### Validating the transcriptome through democratic consensus

Annotating the transcriptome is a difficult and dynamic task, the data and the rules do evolve, and even the most careful manual annotation cannot be expected to provide by itself an incontestable and final truth. There are strengths and weaknesses in each annotation, but good mRNA models supported by strong cDNA data should be found by more than one method. Therefore, an alternative way to select a benchmark may be to take a democratic approach: instead of considering a single track as 'reference', we propose to pool independent cDNA-supported annotations and search for consensual models.

A caveat is that some annotations, such as RefSeq, CCDS or UniProt/SwissProt, are so renowned that most methods use them as a data source in addition to the primary cDNA or protein data, so these models are sticky and will end up validated, whether or not they are correct. Manual annotation is invariably the source of second-hand annotation problems. In fact, in AceView, we do not use SwissProt for this reason, and we now explicitly label all transcripts whose structure is supported only by a RefSeq model as possibly suspect. In the same vein, we use only human cDNAs at the exclusion of any other species, even mammals or primates.

**Figure 3:**
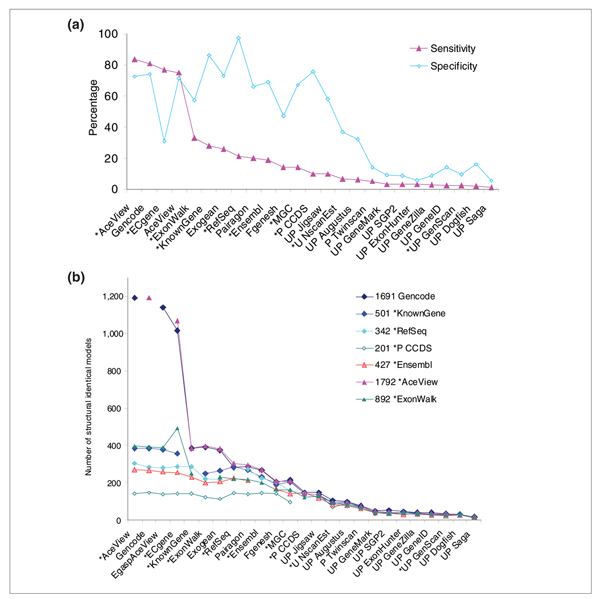
Consensus analysis. **(a) **Sensitivity and specificity at identifying 1,556 consensus transcripts from the pool of the following evidence-based tracks: RefSeq, Known Gene, Ensembl, Gencode, AceView, ECgene and ExonWalk. The sensitivity and specificity of all tracks at identifying these consensus models is plotted and listed in Table 2. **(b)**Closest neighbor consensus, evaluated by switching the track of reference. This figure shows the number of evidence-based models from CCDS, RefSeq, UCSC Known Genes, Gencode, or AceView, ExonWalk and Ensembl whose intron-exon structure is exactly matched by the 25 tracks. Tracks are arranged in decreasing order of averaged detection sensitivity, defined here as the sum of all evidence-based models from these seven reference tracks detected exactly.

We implemented the democratic idea in two flavors in UCSCtrackCompare: we either rotate the reference from Gencode to any other track and perform a closest neighbor consensus analysis (Figure 3b); or, alternatively, we pick as reference a selected pool of tracks and the program extracts their consensual models (for instance those whose intron structure is identical in at least two independent annotation tracks) and measures, for each of the 25 tracks, the number of models exactly matched, hence their sensitivity and specificity.

Table 2 and Figure 3a show the results of the pooling analysis, where the consensual set are the 1,556 spliced transcripts seen by at least 2 of the 7 evidence-based independent methods: Gencode, UCSC Known Genes, RefSeq, Ensembl, AceView, ECgene and ExonWalk. AceView and Gencode fare best, with 84% and 81% sensitivity and 73% and 74% specificity, respectively. The next most sensitive method is ECgene, with 77% confirmed models, but its specificity is only 31%. ExonWalk, UCSC Known Genes, and Exogean provide considerably fewer models (33% to 26% sensitivity). ExonWalk and its closest neighbor ECgene suffer from low specificity, unless both are included in the reference set, probably because they allow combinatorial arrangements of the introns. It would be interesting to know how frequently these entire models are validated in RT-PCR experiments. Then come in order RefSeq, Pairagon, Ensembl, MGC, Fgenesh, and CCDS. Finally, the 12 remaining methods are less sensitive than CCDS, as shown in Figure 3a. This analysis is robust against variations in the composition of the reference set, as long as both Gencode and AceView or ECgene are included. It is even stable if we include the 23 unrelated tracks in the reference pool, yielding 1,957 consensual models (Additional data file 2.6). But if AceView and ECgene are excluded, the consensus falls down to only 478 transcripts across NCBI RefSeq, UCSC Known Gene, EBI Ensembl and Sanger Institute Gencode. In this context, Gencode has an appalling specificity of only 26%. Fortunately, its agreement with AceView cross-validates both methods.

Another view is shown in Figure 3b, which displays the closest neighbor consensus analysis (see also Additional data files 2.6 and 3.1 and 3.6). The sum over seven references of the number of exactly matching models was used to order the tracks. Interestingly, all tracks spontaneously appear to be ordered in overall sensitivity, almost independently of the chosen reference. Gencode and AceView are nearly indistinguishable. With 1,191 models in common, they are by far the most sensitive, but retain a very good specificity. In a robust way, they detect the largest number of models from all other tracks; they are the most inclusive, and three times more thorough than any other reference track (see Additional data file 2.6).

To our surprise, Ensembl, which is often used as a reference catalog, in particular to count the human genes [23], is not consensual, and far below Gencode and AceView in both sensitivity and specificity. While it offers more models than RefSeq (427 versus 342), fewer are confirmed by at least one other method (270 versus 304 in the closest neighbor analysis, 295 versus 332 in the democratic consensus), but the caveat about the artificial increase of specificity and sensitivity of RefSeq certainly applies here. However, the quality of the RefSeq is truly higher than suggested by ExonWalk, Ensembl or Exogean, which validate only 220 to 224 of the 342 RefSeqs: in the EGASP AceView version, we purposely did not use the RefSeqs as a source, yet we confirmed the intron-exon structure of 82% of them (279). However, RefSeq is far from comprehensive. The even smaller CCDS collection is equally well matched by many tracks, but at the protein level (Additional data file 3.6), they are perfectly matched only by Ensembl (201), and not quite by the other members of the CCDS collaboration: RefSeq (197), Gencode (182) and UCSC (189). It is definitely difficult to agree on any standard for protein annotation.

### AceView summarizes GenBank and dbEST into a comprehensive evidence-based gene annotation by performing hand-supervised automatic annotation

The fact that the manually curated Gencode and automatic AceView transcripts are so similar shows that the critical information for the intron-exon structure of a Gencode-like validated annotation is almost entirely contained in the combination of human ESTs, mRNAs and the genome. It appears that AceView is now able to automatically extract this information, with little more noise than a team of careful human experts. The resemblance also indicates that we have the same, possibly biased, way of looking at the data and that we apply similar filters when annotating transcripts.

To reconstruct the genes, AceView considers all cDNA sequences submitted to the public databases, and stringently co-aligns them at their single best position on the genome [7]. Its cDNA to genome alignment algorithms are finely tuned to clip vectors and poly-A and to filter away 3% of the cDNAs because of insufficient quality of their best alignment (especially if they map in multiple genomic locations), and 2.2% because of suspected structural defects; 3.7% of the cDNAs are strand-inverted. The alignments are seeded on exact matches of 15 bp and extended using a finite automaton able to switch from normal to insertion or deletion mode when the EST fasta file starts calling bases at the wrong frequency. Missing exons are researched aggressively, seeding on 6 bp words. Short hits are counted not in base-pairs but in entropy, assuming that each base is statistically independent from its neighbors. This is an over-simplification, but the advantage is that, for instance, an AT rich region is penalized and at the extreme a pure poly-A hit counts zero. The intron-exon boundaries are then refined by co-alignment. Finally, we reject the very long introns unless they are bounded by strong exon support. Aligned cDNAs are then clustered into the minimal set of transcripts (that is, a gene) consistent with their complete intron-exon structure. Most of the gene models with multiple cDNAs have alternative variants, but since September 2004, in order to limit combinatorial expansion of variants, we minimize concatenation by using each cDNA in one and only one transcript, favoring a silent merge in a known compatible transcript, so that only cDNAs containing a specific alternative feature are singled out. As a result, some variants are partial, but 70% of all AceView transcripts have their predicted CDS entirely supported by a single identified cDNA. The remaining models require concatenation of rarer forms, and will possibly be split into multiple alternative variants when additional data become available.

We then name the gene by physical contact to an NCBI Entrez gene model, else by alignment of a RefSeq or GenBank mRNA assigned to an Entrez gene, else by a Pfam-containing name, else by a nickname. The nickname is a number encoded in decodable pseudo English or pseudo Japanese by using a set of phonemes as basic digits. All names and previous aliases are tracked from release to release, and *de facto *AceView closely follows the official HUGO and Entrez gene nomenclature.

However, genome annotation cannot be fully automatic. We must often look at the genes, and take significant decisions to resolve the irregularities. The difficulty is to maintain this hand annotation over the years, as new data become available. For example, the hand annotations of the first *Drosophila *jamboree and of the initial version of the Celera human genome were nearly entirely lost. In AceView, we have limited manpower, just the two of us, so we had to devise an efficient cumulative methodology. Rather than hand annotating the final report of a gene, we only provide hints that are incorporated automatically in the context of the most recent data. For example, if the program seems to merge two genes in an unreasonable way, we do not create a permanent wall between them; instead, we hand annotate a few cDNA clones as having a 'real 3' end' or 'real 5' end', and then port these annotations from build to build. These hints will probably induce the program to split the gene, but if tomorrow a new mRNA sequence strongly bridges the two genes, they will automatically be reunified. When a significant number of genes need the same kind of manual hints, we add a piece of code that performs the same task and then drop the manual annotations once they are automatically reproduced, and even often enhanced. For example, at the EGASP meeting, we learnt that non-standard introns (neither GT/AG, nor GC/AG) are not usually validated by RT-PCR: we now discard any cDNA variant with a nonstandard intron, unless it also brings a novel alternative intron with standard boundaries. As a result, we reduce the noise, but if many clones in a gene use a particular nonstandard intron boundary, for instance because of an error in the genome sequence or because the intron is truly nonstandard, this intron will naturally sift through our tolerant rule and be kept in AceView.

AceView is a service to the community, it does not provide a final answer, but rather some rated proposals aimed at stimulating confirmatory experiments. By using the genome as a guide, it automatically rectifies the sequencing errors in the cDNAs and brings these sequences in line with the excellent quality of the genome itself. But it only provides a partial view of the entire transcriptome, because we are still far from saturation in cDNAs. From release to release, we improve the models by incorporating the latest cDNA data, but also by refining the rules. For example, we recently redefined the gene as a set of transcripts sharing at least one intron boundary, instead of a simple sequence contact. This disentangled the numerous contiguous genes with 3' 5' untranslated region (UTR) overlap and separated the unspliced variants, improving the gene annotation in directions wished for by the users.

Because Gencode annotation is manual, it may prove more difficult for them to include new data or to implement a change in strategy. For example, one can read on the HAVANA guideline site: "Occasionally a short two exon product is supported by Fgenesh and Genscan, in which case the object can be translated. It is then annotated as "believable CDS"." This rule was recently abandoned, but it will be labor-intensive to hand revise all previous models accordingly.

### The puzzle of gene counts

Gencode annotates 3,618 distinct introns. But this is possibly only the tip of the iceberg, since 10,241 other introns in coding regions are predicted in EGASP, mostly by *ab initio *methods. If a proportion of those were correct, we might have mRNA or EST support for maybe only half of the introns and, by extension, we might be missing an appreciable fraction of the genes.

The parallel with the nematode *C. elegans *is interesting. In "So many genes, such a little worm" [24], Hillier *et al*. count 19,735 coding genes in WormBase. But when we analyze all available cDNA sequences in AceView WormGenes [7], we find only 16,094 worm genes with direct experimental evidence, of which about 700 are not annotated in the current WormBase (WS150). There are 12,083 genes supported by cDNAs, mainly from the large scale libraries from Kohara and collaborators (all sequences are in GenBank). An additional 4,011 genes or gene fragments are supported only by the systematic RT-PCR amplification of predicted ORFs from the Vidal ORFeome project [25]. So we conclude that the authors of [24] are confident that, in addition to the cDNA supported genes and the 4,011 gene fragments supported by RT-PCR amplification, close to 4,400 genes that remain pure *ab initio *predictions really exist. Indeed, they exported them to SwissProt/UniProt.

On the other hand, these authors and their collaborators [23] claim that the human genome contains a maximum of 25,000 protein coding genes. They consider that nearly all of them are already known, that the numerous cDNAs that map outside of their official gene list possibly 'reflect reproducible transcriptional noise', and they do not expect any reliable gene to come from *ab initio *predictions. However, AceView unambiguously reconstructs from the readily available human cDNAs about 40,000 genes potentially encoding more than 100 amino acids (22,280 spliced and 18,385 intronless in the August 2005 version), in addition to 13,133 spliced genes encoding shorter proteins or non-coding. Moreover, as we see in EGASP, many *ab initio *predictions can be proposed in between cDNA supported genes and, unlike in the worm, no intense RT-PCR experiments have yet been launched in human. Preliminary EGASP results [6] only provide a lower bound on their existence, because only a fraction of the cDNA supported introns of Gencode and AceView have been validated and *ab initio *predictions are expected to be less expressed. If we apply a uniform method to count genes, we are forced to conclude that human has at least 3 times as many coding genes as the worm, and at least 10 times as many protein isoforms.

## Conclusion

EGASP [5,6] and the availability of the excellent Gencode/HAVANA models have helped us to significantly refine the AceView pipeline. The structure of the AceView transcripts is extremely similar to the Gencode benchmark, so AceView appears to provide today the most comprehensive and accurate representation of the entire human transcriptome. On the other hand, due to a profound lack of experimental protein evidence, annotation of coding sequences remains controversial. We hope that this situation will rapidly improve with the current progress of mass spectrometry and a new understanding of the complex regulation of the translation machinery in vertebrates.

There are currently at least three times more protein coding genes in human than in worm, but the human transcriptome is still far from saturation; 23% of the standard introns observed today in Gencode or AceView are still only supported by a single cDNA. Consistently, the number of variants and alternative introns keeps increasing almost linearly with new cDNA sequences: the addition to GenBank in January 2006 of close to two million 5' complete capped ESTs by the Japanese FLJ group [26] proportionately increased by 26% the number of alternative variants, and added 7% new spliced genes to the AceView collection [7]. In line with these observations, *ab initio *methods propose a wide variety of new models, and suggest that we may currently know only a fraction of the protein coding genes. To learn more about the genes expressed at low level, we depend on future technological improvements, in particular in the microarray domain, and on the continued acquisition of new data. We hope to integrate this flow of information seamlessly in the AceView hand-supervised automatic pipeline.

## Materials and methods

The UCSCtrackCompare program used in this analysis can be downloaded from the AceView web page [7] and can be compiled on any properly configured Unix, Mac or Windows machine. A few precompiled executables and the whole source code are available. A description of the program and relevant examples of the analyses it generates are provided in the four Additional data files.

## Supplementary Material

Additional file 1 Click here for file

Additional file 2 Click here for file

Additional file 3 Click here for file

Additional file 4 Click here for file

## Figures and Tables

**Table 1:** Statistics of the 25 selected tracks, arranged in the order of the UCSC genome browser

UCSC track	Model with introns	Model with introns and CDS	Single exon model (some clipped)	Unique introns in mRNA	All introns in mRNA	Input or method
HAVANA Gencode (Sanger, UK) known + putative	1,691	**649**	70	3,618	9,693	MEP,CA,H
EGASP model submissions						
AceView (NCBI, US)	1,630	1,460	24	3,530	9,597	ME,(H)
UP Dogfish (Sanger, UK)	204	204	15	1,679	1,679	CA
Exogean (ENS, France)	554	538	2	2,855	6,178	MEP,CA
UP ExonHunter (U Waterloo, Canada)	807	807	220	3,237	3,237	MEP,CA
Fgenesh (U London, UK)	462	458	97	2,610	3,241	P,CA
UP GeneId (IMIM, Spain)	267	267	51	1,905	1,905	A
UP GeneMark (Georgia IT, US)	551	551	81	2,185	2,185	A
UP Jigsaw (TIGR, US)	259	259	67	2,168	2,168	MEP,CA
PairagonAny (Wash U, US)	471	437	38	2,300	3,470	MEP?,CA
UP SGP2 (IMIM, Spain)	552	552	159	2,645	2,645	P,CA
P Twinscan-MARS (Wash U,US)	547	547	108	2,501	4,943	CA
UP Augustus Any (U Göttingen, Germany)	312	316	87	2,291	2,291	MEP,CA
UP GeneZilla (TIGR, US)	477	477	179	2,758	2,758	A
UP Saga (UC Berkeley, US)	331	331	47	1,737	1,737	CA
UCSC gene tracks						
*Known Gene (UCSC)	501	477	53	2,264	4,427	MP
*P CCDS	201	201	14	1,296	1,508	MP,H
*RefSeq (NCBI, US)	342	325	41	2,082	2,922	M(E)P,H
*MGC	323	310	19	1,400	2,101	M
*Ensembl (EBI, UK)	427	418	58	2,429	3,548	MEP,CA
*AceView (Aug 2005 NCBI)	1,792	1,627	902	3,812	9,792	ME, (H)
*ECgene (Korea)	3,851	3,551	2,569	3,942	30,660	ME,C
*U NscanEst (Wash U, US)	282	252	27	2,292	2,292	ME,CA
*UP GenScan (MIT, US)	395	395	59	3,042	3,042	A

The number of models, with or without introns (after clipping at region boundaries), the number of spliced coding models, and the number of unique and multiply used introns are given over the 31 ENCODE test regions. Coded information has been added in front of the track name: asterisks distinguish standard gene tracks, available genome-wide, from an ENCODE only track; a U track predicts a unique model per gene; P predicts protein coding regions only. According to their documentation, the programs use different input or methods: M, E, P stand for human mRNA, EST, protein sequences or alignments, respectively; C stands for for conservation, or use of cDNA or protein evidence from other species; A stands for *ab initio *prediction; H stands for Hand curation; and parenthesized letters stand for minimal use of the particular type. Notice the low proportion of Gencode mRNA models with an annotated CDS (in bold).

**Table 2:** Sensitivity and specificity of each method at detecting the 1,556 consensus transcripts

Track	Number of models with introns	Consensual models (of 1,556 total)	Sensitivity	Specificity
*AceView	1,792	1,302	84%	73%
Gencode	1,691	1,255	81%	74%
*ECgene	3,851	1,198	77%	31%
AceView	1,630	1,165	75%	71%
*ExonWalk	892	511	33%	57%
*Known Gene	501	432	28%	86%
Exogean	554	404	26%	73%
*RefSeq	342	332	21%	97%
Pairagon	471	310	20%	66%
*Ensembl	427	295	19%	69%
*MGC	323	217	14%	67%
Fgenesh	462	217	14%	47%
*P CCDS	201	152	10%	76%
UP Jigsaw	259	150	10%	58%
*U NscanEst	282	104	7%	37%
UP Augustus	312	100	6%	32%
P Twinscan	547	77	5%	14%
UP GeneMark	551	50	3%	9%
UP SGP2	552	48	3%	9%
UP GeneZilla	477	47	3%	10%
UP ExonHunter	807	41	3%	5%
UP GeneID	267	38	2%	14%
*UP GenScan	395	37	2%	9%
UP Dogfish	204	33	2%	16%
UP Saga	331	18	1%	5%

Sensitivity and specificity of each method at detecting the 1,556 consensus transcripts across the pool of the following evidence based tracks: RefSeq, Known Gene, Ensembl, Gencode, AceView, ECgene and ExonWalk, as in Figure 3a. Coded information has been added in front of the track name: asterisks distinguish standard gene tracks, available genome-wide, from an ENCODE only track; a U track predicts a unique model per gene; P predicts protein coding regions only.
