# Supplementary material for: AceView: a comprehensive cDNA-supported gene and transcripts annotation
Source: Genome Biol. 2006 Aug 7;7(Suppl 1):S12. doi: 10.1186/gb-2006-7-s1-s12 (PMC1810549; doi:10.1186/gb-2006-7-s1-s12)
Supplement: Additional file 1 [file gb-2006-7-s1-s12-S1.doc]

# The UCSCtrackCompare package: users guide.

# Supplementary material 1 to

# AceView: a comprehensive cDNA-supported gene and transcripts annotation.

Danielle Thierry-Mieg and Jean Thierry-Mieg

[mieg@ncbi.nlm.nih.gov](mailto:mieg@ncbi.nlm.nih.gov)

The UCSCtrackCompare code, written in C, is self documented. This document explains how to use the code, which option can be specified, and which results and analyses are generated.

1. Usage: First download and decompress all the relevant tracks from the UCSC browser (program [UCSCdownload](http://www.ncbi.nlm.nih.gov/IEB/Research/Acembly/Software/UCSCdownload)). Then **edit the two configuration files:** trackConfig.txt and chromConfig.txt. “[trackConfig.txt](http://www.ncbi.nlm.nih.gov/IEB/Research/Acembly/Software/trackConfig.txt)” identifies the reference method (e.g. Havana/Gencode) and lists all the tracks that should be compared to the reference. For each track, you choose a title and give the exact location, on your computer, of the corresponding gff data file. A sample configuration is provided, matching the output of our UCSCdownload.csh script. The “[chromConfig.txt](http://www.ncbi.nlm.nih.gov/IEB/Research/Acembly/Software/chromConfig.txt)” configuration file defines the genomic regions to be analyzed. A sample configuration lists the 31 Encode test regions and the 13 training regions. The 2 configuration files are self-documented.

Unfortunately, we could not find a self contained coordinate file on the UCSC site for hg17, so we read one by one the coordinates of the regions on the UCSC Web browser and copied them in our configuration file. By editing or shortening this table, one can easily run the code on any desired region, for instance a single gene or a single section, which is how we debugged the whole program. All exons outside the selected regions and all introns traversing a boundary are ignored (in the example, these include 127 Gencode transcripts fully outside the ENCODE regions). All exons traversing the boundaries are clipped at the boundary.

2. Executables

A few precompiled executables and the whole source code are available from our web page <http://www.ncbi.nlm.nih.gov/IEB/Research/Acembly/Software>. If you cannot find the proper executable, please email us, we can probably prepare it. If you wish to recompile, please follow the instructions on the Web page. We tested the code on Sun, Alpha, Intel, Opteron, Mac and Windows + Cygwin (http://www.cygwin.com/) and believe it would compile and run on any other 32 or 64 bit Unix/Linux platform equipped with a C compiler.

3. Types of analyses and parameters:

The current program UCSCtrackCompare analyses data in various ways and can produce various types of reports. Of course, all comparisons are strand specific. Ran without parameters, the program will document the available options. There are several classes of parameters:

Configuration parameters

‘-c <file_name>‘ Mandatory, specifies the name of the chromConfig file

‘-t <file_name>‘ Mandatory, specifies the name of the trackConfig file. The Reference track is indicated in that file.

Filters:

‘-cds’ will limit the comparison to the annotated coding parts of the transcripts, trimming out all the declared UTRs;

‘-nosort’ tracks are listed in the output in the order of the trackConfig file. Otherwise, they are sorted according to their score, from best to worst.

‘-aceExport’ exports all the data in .ace format according to the AceView schema, available on our web site. The AceView code can then be used to query and visualize the genes.

## Actions: choose one of the following types of reports:

GLOBAL STATISTICS: ‘-global’, ‘-projectedGlobal’, ‘-global -cds’, ‘-projectedGlobal -cds’: global statistics on all the annotations proposed in the .gff files listed in the trackConfig file pertinent to the regions listed in the chromConfig file. This allows you to verify that the track files are correctly loaded in the UCSCtrackCompare program and gives a first idea about each track.

The output gives a table of global statistics on the selected regions, in global or projected mode, restricted or not to the coding part of the models.

Each table is Excel compatible and has the following self explanatory columns: Program (i.e. track); Nucleotides; Exons; Terminal exons; Introns; mRNA with intron(s); Single exon mRNA; % all mRNA with CDS.

An example of the direct output for the 31 Encode test regions and the 25 selected tracks is provided in the Excel Additional data file 4, page “Summary”, in Table 1, in Additional data file 2 for transcripts, and Additional data file 3 for CDSs.

UCSC REGIONS: ‘-regions’, ‘-regions -cds’: give statistics for each chromosome region defined in the chromConfig file, restricted or not to the coding part.

Each table is excel compatible and has the following self explanatory columns: Program; then each selected region, for example ENm001; ENm002; ENm003; ENm005; ENm007; ENm008; ENm009; ENm010; ENm011; ENm012 etc

An example of the direct output for the 31 test regions followed by 13 training regions and the 25 selected tracks is provided in the Excel Additional data file 4, page “Regions”.

NUCLEOTIDES: ‘-bp’ ‘-bp –cds’ project all the exons on the genome, and count the resulting genome bases covered by the Reference track and the tracks under investigation (true positives: TP), just present in the Reference (false negatives: FN) or just present in the method (false positives: FP). The sensitivity (Sn = TP/ (TP+FN)) and specificity (Sp = TP/ (TP+FP)) are also given.

The output is restricted or not to the coding part, but is always projected: we count the genomic basepairs in the selected regions which are transcribed according to the different program, not the mRNA basepairs. The false positives are split between those from transcripts with introns and those from single exon transcripts, since some programs may prefer to represent the numerous intronless genes represented in GenBank, while others prefer to dust them away, and this influences in an uninteresting way the measures of sensitivity. The program gives both sensitivities but we only discuss sensitivity on spliced models. We also provide for comparison the numbers from tables 4-6 from the Guigo et al. EGASP study [7]. Each table, for the entire models, or limited to the regions annotated as CDS, is excel compatible and has the following self explanatory columns: Program; True positive; False positive in transcript with intron; False positive in intronless transcript; False negative; Sensitivity; Sensitivity eval; Raw specificity; Specificity eval; Specificity, ignoring single exon transcripts, Number bp; Verif (should be 0).

An example of the direct output for the 31 test regions and the 25 selected tracks is provided in the Excel Additional data file 4, page “Nucleotides” and in Additional data file 2 for transcripts, and Additional data file 3 for CDSs (both include comparison to eval [7]).

INTRONS:

‘-projectedIntrons’ and ‘-projectedIntrons -cds’ project all the introns on the genome, consider each unique intron as a pair of coordinates, and compare them.

‘-introns’ and ‘-introns -cds’ is similar, but count each annotated intron separately. The difference with the projected introns case is that a given intron now counts as many times as used in different alternative transcripts. A method which reports a single transcript per gene will fare well in ‘-projectedIntrons’ mode and less well in ‘introns’ mode. A method that chains all the exons in all possible ways will have many false positives in ‘-introns’ mode. The CDS mode analyses introns, restricted to the region annotated as coding.

The output is either in projected mode the #Introns projected, coding or non coding, or # Introns projected, restricted to coding part, in the selected regions, or, in the multiple mode, #Introns multi, coding or non coding, or # Introns multi, restricted to coding part, in the selected regions.

The four outputs are similar, except that the ‘False positive, present in Gencode but over-used’ are counted separately from the other false positives (novel) in the multiple mode. Each table, for the entire models, or limited to the regions annotated as CDS, is excel compatible and has the following self explanatory columns: Program; True positive, identical to Gencode; False positive, present in Gencode but over-used (only in multiple mode); False positive, novel; False negative; Sensitivity; specificity; % missed introns; % wrong introns; All introns; (Verif2; only in the projected mode; should be 0).

.

We also provide in this option an analysis of all unique introns, restricted or not to the region annotated as coding, which are not represented in Gencode, but are found in other tracks. We also show the number represented only in Gencode (or in any other chosen reference). We generate a histogram of the unique introns, not found in the Reference, showing in how many independent tracks each is found. In these histograms, we distinguish those in AceView (this choice is hard-coded, sorry), where we are sure there is good cDNA support, from the others, a priori more hypothetical. This allows us to analyze in depth the eventual missed introns of Gencode, or more generally to rationalize what is outside of Gencode and/or AceView. By touching the source code, one can export selected interesting lists of examples to look at. Please ask [mieg@ncbi.nlm.nih.gov](mailto:mieg@ncbi.nlm.nih.gov) if you wish a built it –verbose option.

An example of the direct output for the 31 test regions and the 25 selected tracks is provided in the Excel Additional data file 4, page “Introns”, Figure 1 and in Additional data file 2 for transcripts, and Additional data file 3 for CDSs (both include comparison to eval [7]).

EXONS:

‘-projectedExons’ ‘-projectedExons -cds’ project all the exons on the genome, consider them as pairs of coordinates, and report again the TP, FN, FP, Sn and Sp, and a few other relevant numbers.

‘-exons’ and ‘-exons -cds’ consider each annotated exon separately. The difference with the projected exons case is that a given exon now counts as many times as used in different alternative transcripts. A method which reports a single transcript per gene will fare well in ‘-projectedExons’ mode and less well in ‘exons’ mode. A method that chains all the exons in all possible ways will have many false positives in ‘-exons’ mode. The CDS mode analyses exons restricted to the region annotated as coding.

The output is either #Exons projected, coding or non coding, or # Exons projected, restricted to coding part, in the selected regions, or in the multiple mode, #Exons multi, coding or non coding, or # Exons multi, restricted to coding part, in the selected regions. The four are similar, except that the ‘False positive, present in Gencode but over-used’ are counted separately from the other false positives in the ‘–exons’ mode. In all cases, the false positive are split between those from transcripts with introns and those from single exon transcripts, since some programs may prefer to represent the numerous intronless genes represented in GenBank, while others prefer to dust them away, and this influences in an uninteresting way the measures of sensitivity. The program gives both sensitivities but we only discuss sensitivity on spliced models. We also it provides for comparison the numbers from tables 4-6 from the Guigo et al. EGASP study [7] when available, that is, in the projected case. Each table, for the entire models, or limited to the regions annotated as CDS, is excel compatible and has the following self explanatory columns: Program , True positive, identical to Gencode; False positive, present in Gencode but over-used (only in multiple mode); False positive, new in multi-exon transcript; False positive, new in single exon transcript; False negative; Sensitivity; (Sensitivity eval, in the projected mode only; this value is canned); Raw specificity; (Specificity eval, in the projected mode only; this value is canned); Specificity, ignoring single exon transcripts; % missed exons; % wrong exons; All exons; (Verif2; only in the projected mode; should be 0).

.We also provide in this option an analysis of all unique exons, restricted or not to the region annotated as coding, which are not represented in Gencode, but are found in other tracks. We also show the number represented only in Gencode (or in any other chosen reference). We generate a histogram of the unique exons, not found in the Reference, showing in how many independent tracks each is found. In these histograms, we distinguish those in AceView (one could choose another test here of course), where we are sure there is good cDNA support, from the others, a priori more hypothetical. This allows us to analyze in depth the eventual missed exons of Gencode, or more generally to rationalize what is outside of Gencode and/or AceView. By touching the source code, one can export selected interesting lists of examples to look at.

An example of the direct output for the 31 test regions and the 25 selected tracks is provided in the Excel Additional data file 4, page “Exons” and in Additional data file 2 for transcripts, and Additional data file 3 for CDSs (both include comparison to eval [7]).

COMPLETE TRANSCRIPT OR PROTEIN MODELS: ‘-transcripts’ and ‘-transcripts -cds’ analyze the whole models. This is the most thorough and most powerful report, because it compares the entire intron-intron structure of the transcripts. Indeed, each spliced transcript is considered as a non-separable chain of introns; single exon transcripts are evaluated separately. Of course, all models count separately: we are in multiple mode, and once the best matching pairs are identified, we compare all alternative variants, as explained in the article. We then report the number of members of best pairs, distinguishing the exactly identical and merely similar transcripts’, the ‘additional variants’, and the transcripts from ‘missed’ or ‘new’ genes. Then in a separate table, we count the introns present in each of these categories of transcripts, distinguishing the TP, FP and FN of different types. One of the interesting uses of this mode is to find the best consensus between many different types of models, taken successively as reference (Table 2 and Figure 3).

Finally, the single exon transcripts are evaluated separately. They are recursively identified as ‘matching intronless transcripts’ if they share a genomic base with a Havana single exon transcript, ‘additional intronless transcripts’ if they share a base with a Havana transcript with introns, or finally as ‘new intronless transcripts’.

The output is either # Intron chaining (all alternative models count) or # Intron chaining (all alternative models count), restricted to CDS, over the selected regions and tracks.

It actually issues 5 different types of analysis per mode, CDS or whole. Each table (whether on entire models, or limited to the regions annotated as CDS), is excel compatible.

The first table counts the transcripts in each method, and examines the percent of edges, introns and exons, in multiple mode. It has the following self explanatory columns: Program; Transcript with intron; Intronless transcript; All transcripts; Exon (in multi-exon transcript, multiple counts, one per alternative); Terminal exons (in multi-exons transcript, multiple counts, one per alternative); Introns (in multi-exons transcript, multiple counts, one per alternative); % Terminal exons in multi-exons transcript, multiple counts, one per alternative.

The second table in the suite performs the actual “Analysis of the transcripts with introns”. Again it splits the transcripts into 4 classes. It provides for each track the following self explanatory columns: Program; Transcript identical to Gencode; Transcript best matching Gencode; New transcript in Gencode gene; New transcript in new gene; Program (more practical to repeat, to generate diagrams in excel!); Missed Gencode transcript in touched gene; Missed Gencode transcript in missed gene; Sensitivity; Specificity; Verif (should be 0).

The third table performs the “Analysis of the Start and Stop among CDS with the same intron structure as the reference”. A difficulty was that unfortunately the format requested by EGASP was not explicit enough, so that half the participants submitted their predicted CDS with and half without the Stop. We tried to fix the problem, as explained in Additional data file 2, but in a few cases, i.e. when the stop is very close or overlaps an intron boundary, our fix is no good. Also note that about one third of Gencode models are partial, so Start here does not necessarily mean Met and End does not necessarily mean Stop.

This analysis provides for each track the following self explanatory columns: Program; Same Start and End; Different start; Different end; Different start and end; Elsewhere (i.e. not overlapping CDS); Reference CDS model with same intron structure; Rate of clearly false (of false end or false start and end or false CDS divided by number of models. We don t include the different starts in this rate).

The fourth table provides the “Analysis of the transcripts without intron”. There are 4 columns: Program; Intronless transcript also found in Gencode; New intronless transcript in Gencode gene; New intronless transcript in new gene.

An example of the direct output for the 31 test regions and the 25 selected tracks is provided in the Excel Additional data file 4, page “Complete Models” and in Additional data file 2 for transcripts and Additional data file 3 for CDSs, and in Figures 2 and 3 and Table 2. The comparative analysis, done in view of analyzing the consensus, and where we switched the reference for spliced transcripts from Gencode to any other main track, is also shown.

MAKE CONSENSUS: ‘-makeConsensus N’: exports in gff format all models whose intron structure is exactly seen in N tracks (we use N=2 or 3). If you then use this gff file as reference, you can measure the sensitivity and specificity of each method relative to their global consensus. See the Excel Additional data file 4, page “Consensus”.

In our opinion, the ‘-introns’ modes are more interesting than the ‘-exons’ modes because there are biological uncertainties and variations on the exact position of the first and last base of a transcript. On the other hand, the intron boundaries are accurate and easy to assess experimentally. This is the reason why, in the ‘-transcripts’ mode, we consider the transcripts as chains of introns rather than chains of exons.

The program output is tab delimited. A good way to visualize the results is to load the output, without modifying it in any way, directly into an Excel spread-sheet. It is then very easy to create colorful tables and diagrams such as those illustrating this paper.

**List of abbreviations**
TP: True positive

FP: False positive

FN: False negative

Sn: sensitivity

Sp: Specificity
