# Supplementary material for: AceView: a comprehensive cDNA-supported gene and transcripts annotation
Source: Genome Biol. 2006 Aug 7;7(Suppl 1):S12. doi: 10.1186/gb-2006-7-s1-s12 (PMC1810549; doi:10.1186/gb-2006-7-s1-s12)
Supplement: Additional file 2 [file gb-2006-7-s1-s12-S2.doc]

# Analyses of the entire transcript models

# Supplementary material 2 to

# AceView: a comprehensive cDNA-supported gene and transcripts annotation.

Danielle Thierry-Mieg and Jean Thierry-Mieg

[mieg@ncbi.nlm.nih.gov](mailto:mieg@ncbi.nlm.nih.gov)

Summary: This document includes notes on the data and the selected tracks, and a series of diagrams showing comparisons, over the 25 tracks, of nucleotides, exons, spliced transcripts and regions of the transcripts annotated as coding. Both qualitative (projected on the genome, each object counts only once) and quantitative (multiple alternative variants contribute separately) analyses are described graphically. Gencode is evaluated, and results from a consensus analysis, depicting the transcripts agreement across all tracks, are presented.

Table of contents:

## [***Notes on the data, the UCSC tracks, and comparison to [6]***](#_The_data,_and_the_UCSC_tracks_compa)

## [***Qualitative (projected) comparison of nucleotides in transcripts***](#nucleotide)

## [***Qualitative and quantitative comparisons of exons in transcripts***](#exons)

## [***Analyses of introns allow to evaluate the completeness of Gencode***](#introns)

## [***Comparison of entire models, mRNAs or proteins, their introns, and their exact coding region***](#_Comparison_of_entire_models_and_the)

## [***Consensus analysis over the annotated CDS***](#consensus)

**In all diagrams**, the data shown constitute a partition, and tracks are ordered from most sensitive to least sensitive. Specificity is less important, because it strongly depends on the size of the target set.

Programs available genomewide, in the UCSC genes tracks, are labeled with an asterisk, *, in front of their names. Programs providing only one model per gene have a U; programs providing only CDS models have a P.

All actual numbers (and more!) are in the corresponding sheets of the excel document, top for entire models, bottom for restriction to CDS (Additional data file 4).

## Notes on the data, the UCSC tracks, and comparison to [6]:

**The data was downloaded Dec14, 2005** from UCSC (on hg17) using the script provided (see Additional data file 1). Caution: the RefSeq track is updated nightly at UCSC. We noticed on January 8 that Augustus EST was actually identical to GeneMark, and dropped Augustus EST from the evaluation. The problem was diligently fixed (Jan 9!) by Kate Rosenbloom at UCSC. We analyzed the confirmed GenMark data from UCSC, but clearly the EGASP group [7] does not use the same data, because we do not agree on only this track in our evaluations of the CDSs (Additional data file 4; their Tables 4 and 5). Gencode submitted on the 44 regions a total of 2,608 known (or reference) transcripts and 156 putative. We were surprised to find that 51 of their submitted models are completely outside the ENCODE regions, and 114 overlap the regions boundaries and had to be clipped (no other EGASP track had this problem). As a reference in our analysis, we used all Gencode transcripts (i.e. 1,651 ‘reference’ transcripts from protein coding genes and 110 ‘putative’ non-coding; from the October 2005 freeze); their ‘pseudogenes’ and ‘artefacts’ were ignored.

We initially **evaluated 19 EGASP tracks and 14 standard genomewide** gene tracks which provide models for the 31 test regions. SPIDA (EBI, UK) and AceScan (Salk Institute, USA) were not used since they only propose exons, not transcripts. The specificities of the programs were extracted from the documentation available on the UCSC genome browser [[3]](http://genome.ucsc.edu/ENCODE/encode.hg17.html), which contains many interesting details and pointers, in particular the credits and bibliography for each track.

Our **annotation in Table 1: “C: uses conservation**” was evaluated from direct examination of gene LOC339766, which is extendable 5’ over 5 exons only if one uses ESTs or mRNAs from Mus musculus, or ab initio predictions. We found that ECgene does not explicitly report use of sequences from other species, yet it appears to use mouse mRNAs/ESTs. On the other hand, Ensembl models in this case refrain from stealing mRNAs from the mouse, just like AceView and other evidence based tracks.

We note that Pairagon and Augustus submitted multiple similar datasets to EGASP, and Ensembl, AceView, Twinscan, SGP, GeneID and Augustus provide both an EGASP set and a public genomewide set. **We removed the less efficient or less meaningful of these near-redundant sets**, i.e. we filtered out PairagonmRNA, PairagonNovel (itself identical to Pairagon Multiple), Ensembl EGASP, AugustusDual, Twinscan Public, SGP Public, Augustus ab initio, Augustus public and GeneID Public. We kept the slightly different public and EGASP sets for AceView, to measure its evolution under the impulse of EGASP. Altogether, we ended up analyzing 15 EGASP tracks and 10 standard genomewide gene tracks.

**To accurately describe the CDS only** (Additional data file 3), we had to solve a technical difficulty, linked to the fact that some methods submitted their CDS including the stop (Gencode, Ensembl, ExonHunter, GeneID, GeneMark, SGP2, Twinscan, and all of the public tracks except ECgene) while most EGASP submitters did not include the Stop, as recommended by the organizers (AceView, Dogfish, Exogean, Fgenesh, Jigsaw, Pairagon, Augustus, GeneZilla, Saga and the public ECgene). This had to be fixed: we systematically added 3 bp to all CDS models, reran the exon analysis and identified all the programs significantly gaining from the addition (the second list above). This systematic correction is used in all our analyses; it only affects the CDS-only results. Guigo et al [7] seem to have done a similar correction. Unfortunately we noticed by looking at a few examples that at least Exogean (ENr122.4.3 and many others), Gencode (AC069356.2-001 or -002), and Ensembl (ENr122.1.7 and 122.1.8) do not use a systematic convention for their inclusion of the Stop codon.

All **statistics over the 31 test regions are reported in the excel document (Additional data file 4)**, in the 4 modes (projected or multiple, whole model or CDS only), exactly as they come from UCSCtrackCompare. Basic numbers are reported below in Table S2.1a (on whole models) and S2.1b (restricted to regions annotated as coding). This paper primarily analyses complete models, while Additional data file 3 deals with the CDS aspects. Guigo et al [7] focused on the projected view of CDSs (part of Additional data file 3) and also, most importantly, on the new protein coding genes and their experimental assessment. Our data are in good agreement on the common analyses (Additional data file 4), nucleotides, exons, in mode projected CDS].

**Table S2.1: Some statistics for transcripts.** **a) Whole models.** The number of models with and without introns, and the numbers of unique and total introns and exons are indicated. **b) Same, but after restricting to the regions annotated as CDS.** Note that Gencode is the only track which fails to annotate proteins in 59% of their transcripts. This clearly biases the comparison at the level of the regions annotated as CDS (see Additional data file 3 for discussion).

| **TABLE S2.1a**  **WHOLE MODELS** Program | mRNA with intron | Single exon mRNA | Unique introns | All introns | Unique exons (some partial) | All exons (some partial) |
| --- | --- | --- | --- | --- | --- | --- |
| Gencode/Havana | 1691 | 70 | 3618 | 9693 | 5585 | 11454 |
| **EGASP MODEL SUBMISSIONS** | | | |  |  |  |
| AceView | 1630 | 24 | 3530 | 9597 | 5273 | 11251 |
| UP Dogfish | 204 | 15 | 1679 | 1679 | 1898 | 1898 |
| Ensembl | 427 | 56 | 2429 | 3548 | 2851 | 4031 |
| Exogean | 554 | 2 | 2855 | 6178 | 3221 | 6734 |
| UP ExonHunter | 807 | 220 | 3237 | 3237 | 4264 | 4264 |
| Fgenesh | 462 | 97 | 2610 | 3241 | 3086 | 3800 |
| UP GeneID | 267 | 51 | 1905 | 1905 | 2223 | 2223 |
| UP GeneMark | 551 | 81 | 2185 | 2185 | 2817 | 2817 |
| UP Jigsaw | 259 | 67 | 2168 | 2168 | 2494 | 2494 |
| Pairagon | 471 | 38 | 2300 | 3470 | 2768 | 3979 |
| UP SGP2 | 552 | 159 | 2645 | 2645 | 3356 | 3356 |
| P Twinscan | 547 | 108 | 2501 | 4943 | 2940 | 5598 |
| UP Augustus | 312 | 87 | 2291 | 2291 | 2690 | 2690 |
| UP GeneZilla | 477 | 179 | 2758 | 2758 | 3414 | 3414 |
| UP Saga | 331 | 47 | 1737 | 1737 | 2115 | 2115 |
| **UCSC GENE TRACKS** | | |  |  |  |  |
| *Known Gene | 501 | 53 | 2264 | 4427 | 2763 | 4981 |
| *P CCDS | 201 | 14 | 1296 | 1508 | 1470 | 1723 |
| *RefSeq | 342 | 41 | 2082 | 2922 | 2360 | 3305 |
| *MGC | 323 | 19 | 1400 | 2101 | 1752 | 2443 |
| *Ensembl | 427 | 58 | 2429 | 3548 | 2853 | 4033 |
| *AceView | 1792 | 902 | 3812 | 9792 | 6796 | 12486 |
| *ECgene | 3851 | 2569 | 3942 | 30660 | 8404 | 37080 |
| *U NscanEst | 282 | 27 | 2292 | 2292 | 2601 | 2601 |
| *UP GenScan | 395 | 59 | 3042 | 3042 | 3496 | 3496 |
| *ExonWalk | 892 | 3 | 2219 | 8057 | 2478 | 8952 |

| **TABLE S2.1b CDS only**  Program | CDS model with intron | Single exon CDS | Unique introns in CDS | All introns in CDS | Unique coding exons (some partial) | All coding exons, some partial |
| --- | --- | --- | --- | --- | --- | --- |
| **Gencode/Havana** | 649 | 74 | 2369 | 5168 | 2768 | 5891 |
| **EGASP MODEL SUBMISSIONS** | | | |  |  |  |
| AceView | 1460 | 194 | 2857 | 7678 | 4155 | 9332 |
| UP Dogfish | 204 | 15 | 1679 | 1679 | 1898 | 1898 |
| Ensembl | 418 | 65 | 2240 | 3260 | 2593 | 3743 |
| Exogean | 538 | 18 | 2336 | 4827 | 2631 | 5383 |
| UP ExonHunter | 807 | 220 | 3237 | 3237 | 4264 | 4264 |
| Fgenesh | 458 | 101 | 2517 | 3063 | 3002 | 3622 |
| UP GeneID | 267 | 51 | 1905 | 1905 | 2223 | 2223 |
| UP GeneMark | 551 | 81 | 2185 | 2185 | 2817 | 2817 |
| UP Jigsaw | 259 | 67 | 2168 | 2168 | 2494 | 2494 |
| Pairagon | 437 | 71 | 2056 | 3145 | 2390 | 3653 |
| UP SGP2 | 552 | 159 | 2645 | 2645 | 3356 | 3356 |
| P Twinscan | 547 | 108 | 2501 | 4943 | 2940 | 5598 |
| UP Augustus | 312 | 87 | 2291 | 2291 | 2690 | 2690 |
| UP GeneZilla | 477 | 179 | 2758 | 2758 | 3414 | 3414 |
| UP Saga | 331 | 47 | 1737 | 1737 | 2115 | 2115 |
| **UCSC GENE TRACKS** | | |  |  |  |  |
| *KnownGene | 477 | 77 | 2075 | 3929 | 2454 | 4483 |
| *P CCDS | 201 | 14 | 1296 | 1508 | 1470 | 1723 |
| *RefSeq | 325 | 57 | 1909 | 2658 | 2192 | 3040 |
| *MGC | 310 | 32 | 1254 | 1909 | 1484 | 2251 |
| *Ensembl | 418 | 67 | 2241 | 3261 | 2596 | 3746 |
| *AceView | 1627 | 1058 | 3106 | 8125 | 5522 | 10810 |
| *ECgene | 3551 | 2854 | 3030 | 24533 | 6938 | 30938 |
| *U NscanEst | 252 | 49 | 2065 | 2065 | 2366 | 2366 |
| *UP GenScan | 395 | 59 | 3042 | 3042 | 3496 | 3496 |
| *ExonWalk | 873 | 22 | 2006 | 7205 | 2334 | 8100 |

**A comparison of sensitivity and specificity for detection of Gencode nucleotides and exons across UCSCtrackCompare and eval, used in Guigo et al [6,**  tables 4-6**]:**

We compared our code, [UCSCtrackCompare](../Thierry-MiegEGASPsupplement1_UCSCtrackCompareusersGuide.doc), to the eval suite used in [6], on what should be exactly identical datasets (projected mode). Numbers are available in Additional data file 4, pages nucleotide and exons. Note that some of their values for standard gene tracks were not considered, because they took the data at UCSC from the hg16 version while we took from the hg17 version. So for instance, instead of AceView Aug05, they compared to the public release then called Acembly, from December 2003, and they used earlier versions of RefSeq or UCSC Known genes. But for all the tracks we report, the measures should be exactly identical. They are very close in CDS-only mode, but for transcripts, they are systematically different in ways that we cannot rationalize. Unfortunately, we were unable to obtain their raw counts, to track down the discrepancies.

In CDS mode (Figure S2.01), except for the measures of GeneMark (sensitivity increased by 2.4% in eval, specificity decreased by 24.8%; we suspect some data mix-up, as explained above) our sensitivity numbers agree within the range of 0.1%-0.64% (average 0.22%). We prefer to compare specificities, ignoring single exon transcripts. But our raw specificity agrees with [6], within 0.47% on average. For exons, the agreement is even better (once removed GeneMark): 0.03% on average for sensitivity, 0.002% for specificity.

The diagrams below show the differences graphically.

**Figure S2.01: Excellent agreement for nucleotides and exons in regions annotated as CDS:** tracks are ordered by sensitivity, from highest to smallest. Corresponding numbers are in Additional data file 4, section nucleotides and exons.

In whole models, including UTR and non-coding transcripts, the agreement is not so good (Figure S2.02). We rechecked all our calculations carefully and cannot see the origin of the difference. Maybe since eval was written for proteins, it has difficulties working on transcript models, especially when there are many alternative, in which an exon can be coding in one variant and not in another. From the documentation, it seems eval does not work directly off the UCSC files: it may have reevaluated the coordinates of the models from the CDS, in a way ‘fixing’ the files in an heterogeneous way.

Systematically, the eval sensitivity on whole transcripts is higher than ours, for all tracks (in nucleotides or exons mode), but less so for ECgene and AceView. The specificity is also different, usually lower, but AceView is much disadvantaged relative to its neighbors, ensembl and Exogean: it looses close to 9 points for nucleotides, and 5 points for exons. This probably does not change the overall picture however, and the global order in the benchmark comparison is very similar.

**Figure S2.02 AceView and ECgene are quite disadvantaged for nucleotides and exons comparisons in whole models, especially relative to Ensembl, Exogean and Pairagon**  (tracks are ordered by sensitivity, from highest to smallest). Other programs, such as Jigsaw, Augustus, ExonHunter or Genscan are also disadvantaged in the mRNA comparison presented in [6], table 6. Corresponding numbers are in Additional data file 4, section nucleotides and exons.

## Qualitative (projected) comparison of nucleotides used in models

**Figure S2.1. Nucleotides found in whole models for transcript, projected, i.e. collapsing alternative variants.**

These results largely contrast with the [same analysis restricted to CDS](../Thierry-MiegEGASPsupplement3_AnalysesRestrictedToCDSs.doc#nucleotidesCDS), where no clear winner is identifiable, as correctly pointed out in [5]. In whole models, AceView and ECgene use the same nucleotides as Gencode (86 to 92% sensitivity) while the known genes, ensembl or RefSeq gene tracks see only 58 to 53% of the Gencode nucleotides respectively. However the public AceView and ECgene suffer from an apparent poor specificity (61 and 43% respectively), while the EGASP AceView has very good specificity (88%). But this is a trivial effect of whether or not the unspliced transcripts are filtered. Indeed, when we separate in yellow the false positive belonging to single exon transcripts from those in red coming from transcripts with introns, we see that most of the apparent specificity defect in the public AceView comes from our choice not to filter single exon transcripts which are well supported by cDNAs.

## Qualitative and quantitative comparison of exons in entire models

Comparisons of introns are presented in the article, figure 1a and b.

**Figure S2.2a: Qualitative comparison of exons or exon fragments in whole models, projected view.**

This view collapses alternative variants and counts exons only once. We distinguish exons exactly found in Gencode (true positive), and among the new exons, those from multiple exons or single exon transcripts. Here again, raw specificity measures in *AceView and *ECgene are dominated by unspliced transcripts, usually from unspliced genes. Specificities calculated on transcripts with introns are more meaningful.

Also note that the contribution of terminal exons often dominates in the “new exon” pile, a drawback of this analysis relative to the corresponding intron diagram, shown in Figure 1 of the paper. Furthermore, one third of the proteins annotated in Gencode are partial, so the effect of terminal exon fragments, each counting once, is important here, and contributes significantly to the red areas in all other tracks.

**Figure S2.2b) Quantitative comparison of exons or exon fragments in whole models, counting separately the alternative variants.**

Same legend as above, except we distinguish an additional false positive category, for exons exactly identical to Gencode exons, but over-used relative to Gencode: this category is prevalent in ECgene and also in ExonWalk, because unlike Gencode or AceView, they prefer to let some concatenation occur in their models rather than leave incomplete transcripts. Like AceView/Acembly was doing two years ago, these programs do not prevent combinatorial as well as Gencode or the current AceView do, but they may turn out to be right. Their hypotheses are testable: can their models be amplified by RT-PCR? Will future cDNAs support those?

For readability, the diagram is clipped at 14,000, although ECgene has more than 37,000 exons (see Additional data file 4, exons). AceView has the best balance of sensitivity and specificity.

## Analyses of introns allow to evaluate the completeness of Gencode

**Figure S2.3. Evaluation of Gencode introns. a) 10365 unique introns are in EGASP but not in Gencode transcripts: The histogram showing how many tracks see those is shown in the figure below.** Numbers are in Additional data file 4, Introns. 8,497 are found only in 1 track and not in Gencode, a majority of those come from ab initio models. Of those, 47 are cDNA-supported in AceView, and probably come from recent cDNAs. However, 681 introns are consensually found in 3 or more tracks, but missing from Gencode. AceView has cDNA support for altogether 340 of these introns (in red). Why not all? Those not seen by AceView or Gencode (in the large range, in blue) are usually matched by RefSeqs or GenBank cDNAs from other mammals (e.g. LOC339766), and may turn out to validate in human (but be expressed at low level). 189 introns are found specifically in Gencode and in no other track.

**Figure S2.3b) (Figure below) Same as 3a, but limited to regions annotated as coding.** Similarly, 31 **introns are found only in Gencode CDS** and no other track, 10,241 are in CDS from other tracks but not Gencode. 863 are consensual (seen by 3 tracks or more); of those, 624 are supported by cDNAs in AceView. Notice that Gencode misses more validated introns in their CDSs than in their mRNAs.

**3a) Zoomed view**

**Figure S2.4. Evaluation of AceView introns. a) 9,505 unique introns are in EGASP but not in AceView CDSs: The histogram showing how many tracks see those is shown in the figure below. If AceView is taken as the reference,** 736 extra introns in CDSs are recovered, and the histogram drops more sharply. Among the missing, 571 are consensual; only a total of 141 are seen by Gencode, and most are unique to Gencode, or rarely seen by other tracks.

**Figure S2.4b) (below) Same as 4a, still with AceView as reference, but considering mRNAs not just CDSs:**

In summary, Gencode sees 8% of the consensual introns missed by AceView, but AceView sees 72% of the consensual introns missed by Gencode. Gencode protein annotation is consensually incomplete. AceView misses less introns, and is more consensual than Gencode at detecting introns, both in CDSs and in whole transcripts. But these differences are relatively marginal; overall, Gencode transcripts, although not their proteins, are of excellent quality.

## Comparison of entire models and their exact coding regions

See Figure 2 in the article for whole models comparisons.

a) In **Figure S2. 5 below, we compare introns after matching the transcripts one to one,** confirming the close resemblance between AceView and Gencode, well above any other track. In this analysis, new genes are distinguished from alternative variants in previously known Gencode gene. Creativity of the ab initio models is easily estimated.

**b) Comparison of** **protein coding regions in whole models. Figure S2.6 below shows the overall excellent agreement in the choice of the coding regions**, once we select only transcripts or models that have the same intron structure.

85% of the structurally identical transcripts agree in their choice of begin and end of the CDS (4363/5126 comparable models), despite the fact that some 31% of the Gencode products are partial, and a handful have plain bugs (see examples in the article). In fact only 9 cases choose the CDS elsewhere in the transcript, in a region with no overlap with the Gencode CDS, and another 57 have overlap, but a different open reading frame. Exogean and ExonWalk have three times more of these anomalies (9% and 10% respectively) than the 10 other top programs (Known genes has only 0.3%, all others are between 3 and 4%; Additional data file 4, Complete models).

But obviously (in pale green), AceView, and to a lesser extent ExonWalk (which uses BESTORF from Softberry), select the Start codon in original ways and often differ from the others. ExonWalk does it in 17% of its models, we do it in 36% of our public models (29% of our EGASP models), on purpose. As outlined in the article, the strategy we apply to choose the Start aims, in the absence of experimental data, at annotating the longest rather than the smallest protein.

1. ***Consensus analysis over the transcripts or entire models***

**a) Table S2.1: Number of consensual transcript models relative to Gencode or AceView taken as reference** (Additional data file 4, consensus). In this closest neighbor analysis, a model whose structure is identical to the selected reference counts as 1. The closest reference is highlighted in blue. Gencode has 12 friendly tracks, mostly among the protein predictors, AceView has 8 friendly tracks, mostly among the evidence based tracks; the total number of models in agreement is slightly larger for AceView; we call it even. But please see Additional data file 3 for a similar table, restricted to CDS, and using ECgene as a reference: Gencode looses each time. In the Gencode/AceView comparison, in CDS mode, Gencode has 1 friendly track (Jigsaw) and AceView 19! There is a problem with Gencode protein predictions.

| **Track** | ***AceView** | **Gencode** |
| --- | --- | --- |
| ***AceView** |  | **1191** |
| **Gencode** | **1191** |  |
| ***ECgene** | **1068** | **1017** |
| ***KnownGene** | **385** | **385** |
| ***ExonWalk** | **396** | **391** |
| **Exogean** | **383** | **377** |
| **Pairagon** | **296** | **286** |
| ***RefSeq** | **304** | **284** |
| ***Ensembl** | **270** | **269** |
| **Fgenesh** | **209** | **205** |
| ***MGC** | **209** | **216** |
| ***P CCDS** | **143** | **149** |
| **UP Jigsaw** | **135** | **147** |
| ***U NscanEst** | **97** | **105** |
| **UP Augustus** | **94** | **99** |
| **P Twinscan** | **74** | **76** |
| **UP GeneMark** | **50** | **50** |
| **UP SGP2** | **40** | **51** |
| **UP GeneZilla** | **37** | **41** |
| **UP ExonHunter** | **39** | **45** |
| **UP GeneID** | **34** | **39** |
| **UP Dogfish** | **29** | **30** |
| ***UP GenScan** | **35** | **34** |
| **UP Saga** | **17** | **18** |
| **total models validated by others** | **5535** | **5505** |

**b) Generalized nearest neighbor consensus analysis:** We present in Figure 3b a study of consensus, using alternatively as reference any of the evidence based tracks: Gencode, UCSC Known Genes, RefSeq, CCDS, AceView, ExonWalk or Ensembl. We examine here how robust is the classification if we use as reference any of the 23 tracks. The EGASP version of AceView, and also NscanEst were not allowed to vote, because they are close relatives of AceView* and Pairagon, so they would be advantaged in a consensual vote. We then order the tracks by the sum of all models validated across the 23 tracks.

This table measures global cross-recognition. Gencode comes first in both sensitivity and specificity; AceView is extremely close; and the classification is very stable when we change the number of voting tracks, except that ExonWalk increases in sensitivity and specificity thanks to ECgene, and Pairagon moves up by Exogean, because it is more often consensual with ab initio predictions. As usual, RefSeq has the best specificity at the consensual game. A similar analysis was done in Additional data file 3 for the regions annotated as CDS.

| **Track** | **Number of models with introns** | **Sum of models validated by the 23 references** | **Number of models validated by the closest independent method** | **Sensitivity relative to the largest consensus** | **Specificity**  **(%)** |
| --- | --- | --- | --- | --- | --- |
| ***AceView** | 1792 | 6629 | 1191 | **100.00%** | **66.5** |
| **Gencode** | 1691 | 6591 | 1191 | **100.00%** | **70.4** |
| **AceView** | 1630 | 6362 | 1139 | **95.60%** | **69.9** |
| ***ECgene** | 3851 | 6381 | 1068 | **89.70%** | **27.7** |
| ***KnownGene** | 501 | 3904 | 385 | **32.30%** | **76.9** |
| ***ExonWalk** | 892 | 3879 | 492 | **41.30%** | **55.2** |
| **Exogean** | 554 | 3450 | 383 | **32.20%** | **69.1** |
| **Pairagon** | 471 | 3486 | 315 | **26.40%** | **66.9** |
| ***RefSeq** | 342 | 3409 | 304 | **25.50%** | **88.9** |
| ***Ensembl** | 427 | 3095 | 270 | **22.70%** | **63.2** |
| **Fgenesh** | 462 | 2758 | 210 | **17.60%** | **45.5** |
| ***MGC** | 323 | 2537 | 223 | **18.70%** | **69** |
| ***P CCDS** | 201 | 2191 | 149 | **12.50%** | **74.1** |
| **UP Jigsaw** | 259 | 2172 | 147 | **12.30%** | **56.8** |
| ***U NscanEst** | 282 | 1488 | 146 | **12.30%** | **51.8** |
| **UP Augustus** | 312 | 1600 | 99 | **8.30%** | **31.7** |
| **P Twinscan** | 547 | 1336 | 79 | **6.60%** | **14.4** |
| **UP GeneMark** | 551 | 907 | 50 | **4.20%** | **9.1** |
| **UP SGP2** | 552 | 848 | 51 | **4.30%** | **9.2** |
| **UP GeneZilla** | 477 | 775 | 45 | **3.80%** | **9.4** |
| **UP ExonHunter** | 807 | 781 | 46 | **3.90%** | **5.7** |
| **UP GeneID** | 267 | 692 | 44 | **3.70%** | **16.5** |
| **UP Dogfish** | 204 | 553 | 37 | **3.10%** | **18.1** |
| ***UP GenScan** | 395 | 704 | 46 | **3.90%** | **11.7** |
| **UP Saga** | 331 | 285 | 18 | **1.50%** | **5.4** |

For comparison, here is the equivalent table using only the 7 references in bold

| **Track** | **Number of models with introns** | **Sum of models validated by the 7 references** | **Number of models validated by the closest independent method** | **Sensitivity relative to the largest consensus** | **Specificity (%)** |
| --- | --- | --- | --- | --- | --- |
| ***AceView** | 1792 | 3880 | 1191 | **100%** | **66%** |
| **Gencode** | 1691 | 3860 | 1191 | **100%** | **70%** |
| EGASP AceView | 1630 | 3723 | 1139 | **96%** | **70%** |
| *ECgene | 3851 | 3617 | 1068 | **90%** | **28%** |
| ***Known Gene** | 501 | 2064 | 385 | **32%** | **77%** |
| ***ExonWalk** | 892 | 1973 | 391 | **33%** | **44%** |
| Exogean | 554 | 1797 | 377 | **32%** | **68%** |
| ***RefSeq** | 342 | 1769 | 304 | **26%** | **89%** |
| Pairagon | 471 | 1695 | 296 | **25%** | **63%** |
| ***Ensembl** | 427 | 1611 | 270 | **23%** | **63%** |
| Fgenesh | 462 | 1280 | 209 | **18%** | **45%** |
| *MGC | 323 | 1189 | 216 | **18%** | **67%** |
| ***P CCDS** | 201 | 996 | 149 | **13%** | **74%** |
| UP Jigsaw | 259 | 928 | 147 | **12%** | **57%** |
| *U NscanEst | 282 | 616 | 105 | **9%** | **37%** |
| UP Augustus | 312 | 613 | 99 | **8%** | **32%** |
| P Twinscan | 547 | 482 | 76 | **6%** | **14%** |
| UP GeneMark | 551 | 301 | 50 | **4%** | **9%** |
| UP SGP2 | 552 | 277 | 51 | **4%** | **9%** |
| UP ExonHunter | 807 | 268 | 45 | **4%** | **6%** |
| UP GeneZilla | 477 | 253 | 41 | **3%** | **9%** |
| UP GeneID | 267 | 219 | 39 | **3%** | **15%** |
| *UP GenScan | 395 | 210 | 35 | **3%** | **9%** |
| UP Dogfish | 204 | 208 | 32 | **3%** | **16%** |
| UP Saga | 331 | 107 | 18 | **2%** | **5%** |

**c) Global consensual analyses, relative to pools:**

The diagram in Figure 3a and table 2 are fairly insensitive to inclusion of more tracks in the pool of reference. In the article, the pool of reference consisted of all evidence based annotation teams: the four annotation groups that tend to make the rule (NCBI RefSeq, UCSC known gene, EBI Ensembl and Sanger Institute Gencode/Havana), and some marginal groups from Korea (ECgene), NCBI (AceView) or UCSC (ExonWalk).

Here we vary the pools of reference, first to including all reference tracks then to including only the four annotation groups (NCBI RefSeq, UCSC known gene, EBI Ensembl and Sanger Institute Gencode/Havana).

1. **Reference: pool of 23 tracks,** all but AceView EGASP and NscanEst: There are **1957 consensual models**, whose exact intron exon structure is seen by multiple tracks.

| **Track** | **Number of models with introns** | **Reference 23: all but AceView egasp and NscanEst** | **Sensitivity23** | **Specificity23** |
| --- | --- | --- | --- | --- |
| ***AceView** | 1792 | 1327 | **68%** | **74%** |
| **Gencode** | 1691 | 1285 | **66%** | **76%** |
| ***ECgene** | 3851 | 1237 | **63%** | **32%** |
| **AceView** | 1630 | 1198 | **61%** | **73%** |
| ***ExonWalk** | 892 | 524 | **27%** | **59%** |
| ***KnownGene** | 501 | 444 | **23%** | **89%** |
| **Exogean** | 554 | 425 | **22%** | **77%** |
| ***Ensembl** | 427 | 424 | **22%** | **99%** |
| ***RefSeq** | 342 | 334 | **17%** | **98%** |
| **Pairagon** | 471 | 330 | **17%** | **70%** |
| **Fgenesh** | 462 | 273 | **14%** | **59%** |
| ***MGC** | 323 | 237 | **12%** | **73%** |
| **UP Jigsaw** | 259 | 199 | **10%** | **77%** |
| ***P CCDS** | 201 | 190 | **10%** | **95%** |
| **P Twinscan** | 547 | 152 | **8%** | **28%** |
| **UP Augustus** | 312 | 151 | **8%** | **48%** |
| ***U NscanEst** | 282 | 114 | **6%** | **40%** |
| **UP GeneMark** | 551 | 99 | **5%** | **18%** |
| **UP SGP2** | 552 | 91 | **5%** | **16%** |
| ***UP GenScan** | 395 | 84 | **4%** | **21%** |
| **UP GeneZilla** | 477 | 80 | **4%** | **17%** |
| **UP ExonHunter** | 807 | 79 | **4%** | **10%** |
| **UP GeneID** | 267 | 75 | **4%** | **28%** |
| **UP Dogfish** | 204 | 47 | **2%** | **23%** |
| **UP Saga** | 331 | 24 | **1%** | **7%** |

**2- Reference 2: the four ‘official’ centers, NCBI RefSeq, UCSC known gene, EBI Ensembl and Sanger Institute Gencode/Havana. There are only 478 consensual (N=2) models**.

| **Track** | **Number of models with introns** | **Reference: 478 models from the 4 big** | **Sensitivity**  **4 big** | **Specificity**  **4 big** |
| --- | --- | --- | --- | --- |
| **Gencode** | 1691 | 437 | **91%** | **26%** |
| ***AceView** | 1792 | 420 | **88%** | **23%** |
| **AceView** | 1630 | 413 | **86%** | **25%** |
| ***KnownGene** | 501 | 411 | **86%** | **82%** |
| ***ECgene** | 3851 | 382 | **80%** | **10%** |
| ***RefSeq** | 342 | 323 | **68%** | **94%** |
| **Exogean** | 554 | 292 | **61%** | **53%** |
| **Pairagon** | 471 | 287 | **60%** | **61%** |
| ***Ensembl** | 427 | 280 | **59%** | **66%** |
| ***ExonWalk** | 892 | 262 | **55%** | **29%** |
| **Fgenesh** | 462 | 208 | **44%** | **45%** |
| ***MGC** | 323 | 196 | **41%** | **61%** |
| ***P CCDS** | 201 | 148 | **31%** | **74%** |
| **UP Jigsaw** | 259 | 136 | **28%** | **53%** |
| ***U NscanEst** | 282 | 92 | **19%** | **33%** |
| **UP Augustus** | 312 | 90 | **19%** | **29%** |
| **P Twinscan** | 547 | 67 | **14%** | **12%** |
| **UP GeneMark** | 551 | 41 | **9%** | **7%** |
| **UP SGP2** | 552 | 40 | **8%** | **7%** |
| **UP GeneZilla** | 477 | 39 | **8%** | **8%** |
| **UP ExonHunter** | 807 | 33 | **7%** | **4%** |
| **UP GeneID** | 267 | 32 | **7%** | **12%** |
| ***UP GenScan** | 395 | 32 | **7%** | **8%** |
| **UP Dogfish** | 204 | 30 | **6%** | **15%** |
| **UP Saga** | 331 | 14 | **3%** | **4%** |

We have lost the big discovery of the EGASP project by restricting to the 4 ‘official’ RefSeq, UCSC Known genes, Ensembl and Gencode: the large increase in number of confirmed transcript models. Notice that Gencode and AceView are still the most sensitive, but they become disgustingly non-specific. ExonWalk and ECgene, who support each other, move down most in this diagram relative to the pool of 7 evidence-based methods. Annotation of the transcriptome is much improved if the efforts of all are taken into account when selecting the consensus.

3- **Reference 3: Non evidence based consensual models: (mainly, ab initio component). The reference here is the pool of all 23 minus the pool of the 7 evidence based. There are altogether 401 consensual models in this category.**

This analysis gives a sense of the consensus across ab initio model-makers. This subtracted set of transcripts, seen at least twice among the 23 tracks, but not seen twice by the 7 evidence based mRNA annotators, corresponds to consensual predictions not yet supported by cDNA evidence. Those could be the first to test by RT-PCR, but one should be aware (as we are not) of the biases introduced by the filiation of the prediction programs, since that may seriously affect the consensus. Some strong resemblance between Ensembl and Twinscan is apparent. One should not be surprised to see Ensembl top this list: they announce that there is a good ab initio component in their models; we see that represents actually close to one third of their models, and most are consensual with another ab initio program. Note that the 38 CCDS in this set most likely belong to mRNAs with introns outside the CDS. Because the set of 7 evidence based all provide mRNA, not protein models, these CCDS were not consensual in the set of 7 (probably their mRNA structure was).

| **Track** | **non-evidence**  **based** | **Sensitivity**  **23minus7** | **Specificity**  **23minus7** |
| --- | --- | --- | --- |
| ***Ensembl** | 129 | **32%** | **30%** |
| **P Twinscan** | 75 | **19%** | **14%** |
| **Fgenesh** | 56 | **14%** | **12%** |
| **UP Augustus** | 51 | **13%** | **16%** |
| **UP Jigsaw** | 49 | **12%** | **19%** |
| **UP GeneMark** | 49 | **12%** | **9%** |
| ***UP GenScan** | 47 | **12%** | **12%** |
| **UP SGP2** | 43 | **11%** | **8%** |
| ***ECgene** | 39 | **10%** | **1%** |
| ***P CCDS** | 38 | **9%** | **19%** |
| **UP ExonHunter** | 38 | **9%** | **5%** |
| **UP GeneID** | 37 | **9%** | **14%** |
| **UP GeneZilla** | 33 | **8%** | **7%** |
| **AceView** | 33 | **8%** | **2%** |
| **Gencode** | 30 | **7%** | **2%** |
| ***AceView** | 25 | **6%** | **1%** |
| **Exogean** | 21 | **5%** | **4%** |
| **Pairagon** | 20 | **5%** | **4%** |
| ***MGC** | 20 | **5%** | **6%** |
| **UP Dogfish** | 14 | **3%** | **7%** |
| ***ExonWalk** | 13 | **3%** | **1%** |
| ***KnownGene** | 12 | **3%** | **2%** |
| ***U NscanEst** | 10 | **2%** | **4%** |
| **UP Saga** | 6 | **1%** | **2%** |
| ***RefSeq** | 2 | **0%** | **1%** |
