# Supplementary material for: AceView: a comprehensive cDNA-supported gene and transcripts annotation
Source: Genome Biol. 2006 Aug 7;7(Suppl 1):S12. doi: 10.1186/gb-2006-7-s1-s12 (PMC1810549; doi:10.1186/gb-2006-7-s1-s12)
Supplement: Additional file 3 [file gb-2006-7-s1-s12-S3.doc]

# Analyses limited to the regions annotated as protein-coding

# Supplementary material 3 to

# AceView: a comprehensive cDNA-supported gene and transcripts annotation.

Danielle Thierry-Mieg and Jean Thierry-Mieg

[mieg@ncbi.nlm.nih.gov](mailto:mieg@ncbi.nlm.nih.gov)

Summary: This document follows Additional data file 2, but the analysis is limited to the regions annotated as CDS. When alternative variants are considered separately, the resemblance between Gencode and AceView remains apparent. But when alternative variants are collapsed, as done in [7], the amazing coherence between Gencode and AceView is obscured. As shown in our consensus analysis, this bias is due to the fact that Gencode annotates proteins in only 41% of their mRNAs.

Table of contents:

## [***Comparing CDSs is comparing opinions, not facts.***](#_Comparing_CDSs_is_comparing_opinion_1)

## [***Qualitative (projected) comparison of nucleotides in CDS***](#_Qualitative_(projected)_comparison__1)

## [***Qualitative and quantitative comparisons of introns in CDS***](#_Qualitative_and_quantitative_compar_2)

## [***Qualitative and quantitative comparisons of exons in CDS***](#_Qualitative_and_quantitative_compar_3)

## [***Comparison of whole CDS models, and their exact coding region***](#_Comparison_of_whole_CDS_models,_the)

## [***Consensus analysis over the annotated CDS***](#_Changing_the_reference_sample:_Cons)

**In all diagrams**, the data shown constitute a partition, and tracks are ordered from most sensitive to least sensitive. Specificity is less important, because it strongly depends on the size of the target set.

Programs available genomewide, in the UCSC genes tracks, are labeled with an asterisk, *, in front of their names. Programs providing only one model per gene have a U; programs providing only CDS models have a P.

All actual numbers (and more!) are in the corresponding sheets of the excel document, top for entire models, bottom for restriction to CDS [Additional data file 4].

## Comparing CDSs is comparing opinions, not facts:

Such comparison is dangerously biased, since CDSs are all predicted, and not validated experimentally. Furthermore, the bias on CDS-only analyses may be important, because Gencode predicts hypothetical proteins in only 41% of its models. Let us illustrate the problem: Gencode proposes a total of 1,691 transcript models. The public AceView proposes 1,792 transcripts: 1,191 (70.4%) are structurally identical to Gencode models; both sensitivity and specificity are surprisingly high. But if we now clip the UTRs of all models, we discard 1,042 Gencode models with no annotated CDS, and are left with only 649 Gencode CDS models with introns, while the same treatment on AceView transcripts still has 1,460 CDS models with introns, with 460 structurally identical to Gencode (still the same proportion of the Gencode CDSs, 70.9%, but a very low proportion of the AceView CDSs, 31.5%). While Gencode and AceView transcripts are extremely similar, AceView would seem highly non-specific, but are we measuring something meaningful here?

In fact, although this is not true for their excellent transcripts annotation (Additional data file 2), Gencode protein annotation is consensually incomplete (see paragraph [6](#consensus) below, and Additional data file 2).

To best illustrate this point, we can compare each track to Gencode or ECgene taken as reference, and count how many of their CDS models have the very same intron structure. Table S3.1: shows the results of the vote: in absolutely all cases, ECgene wins. Similarly AceView wins in 19 of 20 cases in the same test. Any CDS annotation, including Gencode’s, remains questionable: in the absence of data, nobody should believe they have the final answer.

Table S3.1 Number of models of identical structure annotated as CDS by ECgene or Gencode and any other track.

| **Track** | ***ECgene** | **Gencode** |
| --- | --- | --- |
| ***ECgene** |  | **476** |
| **AceView** | **849** | **468** |
| ***AceView** | **824** | **459** |
| ***ExonWalk** | **534** | **323** |
| **Gencode** | **476** |  |
| ***KnownGene** | **357** | **328** |
| **Exogean** | **357** | **308** |
| **Pairagon** | **327** | **267** |
| ***RefSeq** | **285** | **263** |
| ***Ensembl** | **277** | **256** |
| **Fgenesh** | **243** | **214** |
| ***MGC** | **229** | **193** |
| ***P CCDS** | **188** | **182** |
| **UP Jigsaw** | **186** | **178** |
| ***U NscanEst** | **120** | **116** |
| **UP Augustus** | **115** | **110** |
| **P Twinscan** | **103** | **88** |
| **UP GeneMark** | **60** | **50** |
| **UP ExonHunter** | **61** | **53** |
| **UP GeneZilla** | **56** | **45** |
| **UP SGP2** | **54** | **49** |
| **UP Dogfish** | **47** | **39** |
| ***UP GenScan** | **46** | **35** |
| **UP GeneID** | **43** | **37** |
| **UP Saga** | **24** | **21** |
| **sum of models validated by other tracks** | **5861** | **4558** |

## Qualitative (projected) comparison of nucleotides in CDS

**Figure S3.1. Nucleotides found in region annotated as coding, projected, i.e. counted only once per base position and collapsing alternative variants**. [Unlike in transcripts](../Thierry-MiegEGASPsupplement2_TranscriptsAnalyses.doc#nucleotide), there is little difference across tracks in sensitivity. Specificity is reduced in large part because Gencode annotates CDSs in only 41% of its transcripts, and because of intronless transcripts (in yellow).

## Qualitative and quantitative comparisons of introns in CDS (To be compared to Figures 1a and 1b of the article, for entire models.)

**Figure S3.2a: Qualitative comparison of introns: projected view, collapsing alternative variants**

**Figure S3.2b: Quantitative comparison of introns: alternative variants count individually**

**In the next page, same figure zoomed** (ECgene is truncated): Exogean might be considered the best in this view, in combined sensitivity and specificity (but CDS views are biased)

## Qualitative and quantitative comparisons of exons in CDS

**a) Figure S3.3a: Qualitative comparison of exons or fragments in CDS: projected view, collapsing alternative variants.**

All UTRs were clipped. Remember that one third of the proteins annotated in Gencode are partial, so the effect of terminal exon fragments, each counting once, is not negligible here, and contributes significantly to the red area. Same warning as before, exons exactly identical in Gencode and other methods, such as AceView, turn from green to red, because close to 60% of Gencode transcripts have no annotated CDS (see Additional data file for the comparable diagrams on entire models)

**b) Figure S3.3b: Quantitative comparison of exons in CDS: alternative variants count individually.** Jigsaw might be considered the best in the projected view and Exogean in the quantitative view, in combined sensitivity and specificity (but CDS views are biased: the fact that Gencode annotated CDSs in only 41% of their transcripts leads to over-estimation of the false negatives).

Same figure, zoomed (ECgene is truncated)

## Comparison of whole CDS models, and their exact coding region

**Figure S3.4 shows the region of the models annotated as CDS that have the exact same intron structure as the Gencode CDS.** It should be compared to Figure 2 of the article, for a more accurate comparison, at the level of the transcripts. In this important view, Known Genes (at UCSC) might be considered the best for combined sensitivity and specificity (but CDS views are biased!).

##

**Zoomed view:**

**b)** **Comparison of protein coding regions in CDS mode (UTR removed before hand). Figure S3.5** **shows the overall excellent agreement in the choice of the coding regions,** once we select only CDS that have the same intron structure, with the exception of the choice of start in AceView and ExonWalk, which do not follow the current consensus, as explained in Additional data file 2. The red (different start and end) are in part due to the few mis-annotated proteins in Gencode, examples of which are cited in the article.

## Changing the reference sample: Consensus analysis over the annotated CDS

We examine here the number of CDS models (once UTRs are clipped) which have the exact same intron structure as models from any other track, in a closest neighbor analysis. We order the 23 tracks by the sum of their maximum number of validated CDS models (the encode AceView and Twinscan were not allowed to vote, because they are close relatives of AceView* and Pairagon, so they would be advantaged in a consensual vote; all data are in Additional date file 4, consensus). This table measures global cross-recognition. ECgene is number 1 in this analysis. For sensitivity, Gencode comes only in fourth position, after ECgene, AceView, and ExonWalk. This time, CCDS not RefSeq has the best specificity (100 %!) at the consensual game.

Table S3.2: Consensual analysis N x N (23 x 25) of the CDS, by mutual agreement.

| **Track** | **Number of CDS models with introns** | **Sum of CDS models validated by the 23 references** | **Number of models validated by the closest independent method** | **Sensitivity relative to the largest consensus** | **Specificity**  **(%)** |
| --- | --- | --- | --- | --- | --- |
| ***ECgene** | 3551 | 5716 | 824 | **97.10%** | **23.2** |
| **AceView** | 1460 | 5417 | 849 | **100.00%** | **58.15** |
| ***AceView** | 1627 | 5336 | 824 | **97.10%** | **50.65** |
| ***ExonWalk** | 873 | 4107 | 534 | **62.90%** | **61.17** |
| **Gencode** | 649 | 4450 | 476 | **56.10%** | **73.34** |
| ***KnownGene** | 477 | 4043 | 359 | **42.30%** | **75.26** |
| **Exogean** | 538 | 3595 | 357 | **42.00%** | **66.36** |
| **Pairagon** | 437 | 3674 | 327 | **38.50%** | **74.83** |
| ***RefSeq** | 325 | 3574 | 285 | **33.60%** | **87.69** |
| ***Ensembl** | 418 | 3426 | 277 | **32.60%** | **66.27** |
| **Fgenesh** | 458 | 3093 | 243 | **28.60%** | **53.06** |
| ***MGC** | 310 | 2718 | 229 | **27.00%** | **73.87** |
| ***P CCDS** | 201 | 2688 | 201 | **23.70%** | **100** |
| **UP Jigsaw** | 259 | 2596 | 186 | **21.90%** | **71.81** |
| ***U NscanEst** | 252 | 1867 | 155 | **18.30%** | **61.51** |
| **UP Augustus** | 312 | 1803 | 115 | **13.50%** | **36.86** |
| **P Twinscan** | 547 | 1578 | 103 | **12.10%** | **18.83** |
| **UP GeneMark** | 551 | 978 | 60 | **7.10%** | **10.89** |
| **UP ExonHunter** | 807 | 916 | 61 | **7.20%** | **7.56** |
| **UP GeneZilla** | 477 | 881 | 56 | **6.60%** | **11.74** |
| **UP SGP2** | 552 | 940 | 54 | **6.40%** | **9.78** |
| **UP Dogfish** | 204 | 654 | 47 | **5.50%** | **23.04** |
| ***UP GenScan** | 395 | 746 | 46 | **5.40%** | **11.65** |
| **UP GeneID** | 267 | 731 | 44 | **5.20%** | **16.48** |
| **UP Saga** | 331 | 345 | 24 | **2.80%** | **7.25** |
